# Biohybrid chiral materials for an ultralight reconfigurable flying robot

**DOI:** 10.1126/sciadv.aef0912

**Published:** 2026-07-23

**Authors:** Qi Yang, Bingnan Zhou, Rahul Chand, Duy Huynh, Fahad Ahmed Korai, Jianfeng Yang, Wan Shou, Ignazio Maria Viola, Dengfeng Li, Hao Zeng

**Affiliations:** ^1^ State Key Lab of Advanced Optical Polymer and Manufacturing Technology, Key Lab of Rubber-Plastics, Ministry of Education/Shandong Provincial Key Lab of Rubber-plastics, School of Polymer Science and Engineering, Qingdao University of Science and Technology, Qingdao 266042, China.; ^2^ Light Robots, Faculty of Engineering and Natural Sciences, Tampere University, Tampere 33720, Finland.; ^3^ Department of Mechanical Engineering, University of Arkansas, Fayetteville, AR 72701, USA.; ^4^ School of Engineering, Institute for Energy Systems, University of Edinburgh, Edinburgh, EH9 3FB, UK.; ^5^ Department of Industrial Engineering, Alma Mater Studiorum University of Bologna, Forlì, 47121, Italy.

## Abstract

Structural handedness plays a key role in governing various motions in animals and plants, especially for rotational motion and aerodynamic response in flying systems. However, harnessing handedness in artificial materials to enable dynamic, wireless control of untethered miniature flyers remains largely unexplored. Here, we report a feather-polymer hybrid chiral flyer that achieves light-controlled airborne motion. The device integrates natural afterfeathers with a light-responsive actuator synthesized via photopolymerization of azobenzene-functionalized groups, enabling programmable and reversible photomechanical actuation. The handedness of the flyer arises from actuator-induced torsion, while optical modulation of aerodynamic drag allows controlled spinning and altitude adjustment under steady airflow. We demonstrate independent control of multiple untethered flyers and midair collection through vortex-induced aggregation. Computational fluid dynamics simulations reveal asymmetric pressure gradients that are associated with the direction of rotation, while localized low-airflow regions facilitate aerial trapping. These findings introduce a strategy for programmable, wireless ultralight flyers, bridging natural structures and synthetic actuators toward intelligent airborne systems.

## Teaser

Light-responsive polymer strips enable control of feather spin and flight in wind.

## INTRODUCTION

Micro aerial vehicle (MAV) is a nascent research frontier aiming to develop miniature untethered objects with controllable movement in the air (*1*–*3*). A passive flyer is an unpowered aerial vehicle or object that relies solely on environmental forces such as winds, gravity, or thermal currents for flight, without active propulsion systems (*4*, *5*). The main mechanism is the implementation of a structure with effective aerodynamic interaction with the air that generates lift and drag to sustain the gravitational force and sometimes governs the direction of the gliding flight (*6*). Compared to the active flyers, such as vehicles based on rotary propellers (*7*, *8*) and flapping wings (*9*), and insects or birds found in biological kingdoms, the passive ones are often observed in plant species which show two notable merits. The first one is energy efficiency for airborne transportation (*10*). For instance, the maple samara generates a stable leading-edge vortex (*11*) that critically enhances the aerodynamic efficiency for extended suspension and long-distance travel using crosswinds. The second one is the material’s intrinsic sensitivity in the wind dispersal property. As exemplified by dandelion seed shape morphing, the pappus can open or close due to hygroscopic deformation, which consequently modulates air drag automatically by varying the air moisture (*12*, *13*). Nature inspiration has driven the realization of biomimetic flyers, imitating the aerodynamics, flight trajectories, and stimuli responsiveness observed in the biological kingdom (*5*, *14*–*16*). These developments have triggered novel untethered control of midair devices (*17*), together with a new concept of distributed sensors (*4*, *18*–*21*).

The way for replicating the natural design into man-made micro aircraft is straightforward. The two main categories in wind-dispersed diaspores (*22*), i.e., pappus-bearing seeds and winged seeds, suggest two design principles for attaining the high aerodynamic efficiency. The first principle is the high porosity. In dandelion, each seed is topped with a crown of fine bristles, forming a centrally symmetric porosity gradient. Air layers form around each filament interfere with one another (the wall effect), notably reducing the airflow passing through the structure, bringing the formation of a stable separated vortex ring (SVR), which is associated with enhanced air drag and ensures the airborne stability (*23*). This porosity-enhanced dispersal is observed in *Taraxacum officinale* species (dandelion) that exhibit central symmetry in configuration, as well as poplar seeds, which consist of randomly distributed cotton-like fibers. The second principle is the autorotation mechanism (*5*). This case often adopts the design of a planar wing with highly asymmetrically distributed mass, where the seed base stands on one edge of the wing, shifting the centroid to one side. The mass asymmetry causes the autorotation during the descent, which is a self-stabilizing behavior resulting from a finely balanced interplay between gravity, inertia, and aerodynamic forces (*5*, *20*). On the basis of the above, a few key messages are conveyed to the structural designers: Porosity enhances drag, structural symmetry induces airborne stability, and asymmetry is responsible for motion chirality, specifically spinning with a certain handedness. The general goal for biomimetic flight robotics is not only to reproduce the same aerodynamic efficiency of the biological ones but also to explore pathways to untethered control the aerodynamics on purpose.

Recently, a game changer came along with a combination between the passive airborne structure and active stimuli-responsive polymers (*16*, *24*). The responsive polymers are synthetic materials capable of large deformation upon external stimuli (*25*). Among them, responsive polymer thin films provide a feasible pathway to deform the flyer configuration and consequently tune the aerodynamic property through the stimulus that is sensed from the environment (e.g., moisture) (*26*) or delivered through long-distance such as light beam irradiation (*27*). Introducing untethered controllability into aerodynamic structures requires ultralight weight and efficient actuation. The former refers to a necessity that the material has a relatively low mass portion of the entire flyer but still can efficiently change the morphing bearing the aerodynamic drag; the latter indicates relatively high responsive speed compared to the airborne motion period and reliable reversibility during the cyclic actuation. To date, there are several materials fulfilling these requirements. They are, e.g., photothermally responsive liquid crystalline elastomer (*28*, *29*), photochemically reacting liquid crystalline network (LCN) (*30*), and functional bilayers (*31*). Pioneering examples have showcased the take-off/landing action under a steady wind flow influenced by light illumination (*15*, *16*), light steering of gliding direction (*24*), and sensor function enabled by humidity-induced color change (*32*).

The diversity of midair maneuvering is accounted to the control of vertical movement (take-off and landing), two-dimensional (2D)–to–3D navigation, and rotating with controlled handedness. The last one has not yet been reported. From the perspective of a minimally controlled midair robot, five degrees of freedom are required: the 3D spatial coordinates (*X*, *Y*, and *Z*) and the body orientation, defined by the polar angle (θ) and azimuthal angle (φ). Previous studies have demonstrated the potential for controlling 3D coordinates using a dandelion-inspired model (*15*); however, this system relies on SVR for stabilization, making it unsuitable for rotational control. In contrast, a maple seed–inspired model (*24*) remains airborne through continuous autorotation, but this rotation cannot be halted, preventing the maintenance of a fixed orientation (φ) during flight. Critically, neither system allows active modulation of rotational state, such as turning rotation on or off or switching between left- and right-handed rotation during free flight. As a result, a key degree of freedom, namely, the azimuthal orientation (φ), remains unaddressed.

Here, we attempt to explore the aforementioned challenge by reporting a biohybrid flying structure weighing only about 1 mg that exhibits optically tuning in rotational speed in the air. Different midair dances, i.e., optical switching between take-off and landing and left-handed and right-handed spinning, have been enabled by using different mode LCN strip actuators. In addition, we also demonstrate the individual control of multiple flyers by projected light patterns and flyer harvesting using vortex-induced negative pressure.

## RESULTS

### System design

**Fig. 1.**
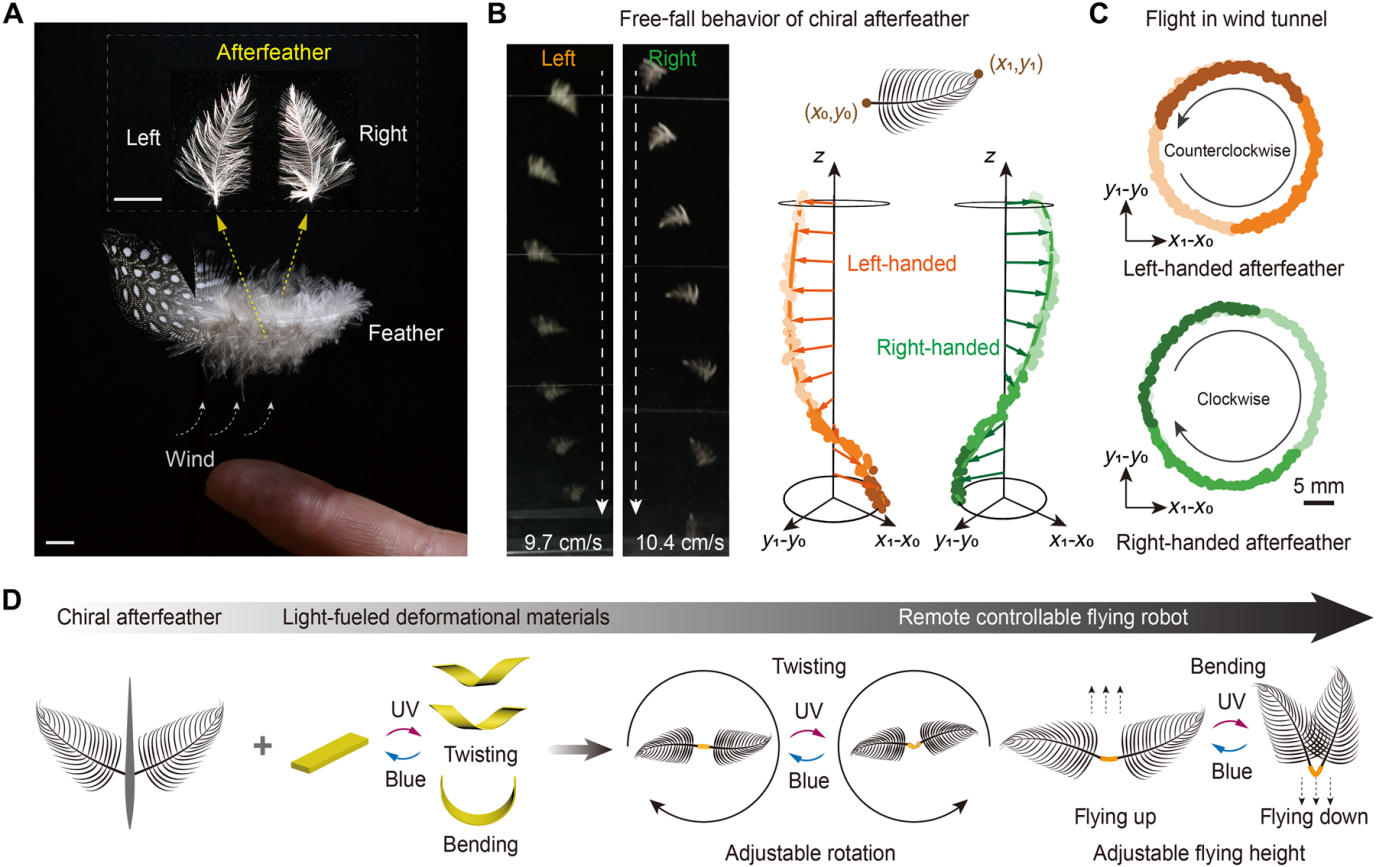
Design concept of feather robotic assembly based on morphing. (**A**) Photographs of left and right afterfeathers from a floating feather. Scale bar, 5 mm. (**B**) In the free-fall experiment, the left and right afterfeathers exhibit opposing handedness in rotation: counterclockwise and clockwise, respectively. (**C**) When flying in wind tunnel, left-handed afterfeather spins counterclockwise and right-handed afterfeather spins clockwise. (**D**) Light-controlled gliding robotic assemblies from chiral afterfeathers and deformable materials, featuring controllable gliding rotation and height regulation.

Feather, a branching structure grows from birds’ skin, are one of the lightest compartments found in biology at the macro scale (*33*). If one observes in detail the structure, for instance, a feather taken from a guinea fowl as shown in Fig. 1A, it consists of a central shaft and branches of hair-like barbs on either side forming a thin layer of vane (see details in fig. S1). The shaft is hollow in nature, while the barbs have high porosity, in total providing ultralight weight and ultrahigh aerodynamic efficiency, which are crucial for flight. Among the hierarchy layers, the tinniest filament is called the afterfeather, which emerges from the underside of the shaft. This afterfeather has high softness as well as porosity, essential for an animal’s thermal insulation and generating aerodynamic drag during flight. A detailed structural analysis of natural afterfeathers and the variability across natural samples are provided in note S1. There are two merits particularly intriguing for the design of MAVs. First, the afterfeathers are grown with certain handedness. As shown from the insets in Fig. 1A, a left-handed afterfeather (directing from the tip to the root) and right-handed ones can be collected from different sides of the feather. Second, the descent speed in the steady airflow is ultralow. Figure 1B shows the snapshots of two free-falling afterfeathers with both left- and right-handed types, presenting the descent velocity around 0.12 ± 0.02 m/s (0.50 ± 0.05 m/s for dandelion seed and 0.40 ± 0.02 m/s for the entire feather; *n* = 10 samples). These two merits indicate an ultrahigh aerodynamic efficiency in the naturally evolved structure and chirality that can be used to attain diverse modes in midair flight. It is worth noting that the comfort range of indoor wind speeds is 0.1 to 0.2 m/s, according to International Organization for Standardization standards. Afterfeathers are uniquely suited for creating steerable flyers within typical indoor environments, without the need for externally imposed or perceptible airflow. In contrast, other natural systems, such as dandelion and maple seeds, as well as previously reported artificial flyers, operate at higher wind speeds.

Figure 1B (right) shows the tracking of the trajectory during the free descent inside steady air, in which (*x*_0_, *y*_0_) and (*x*_1_, *y*_1_) indicate the root and tip position, respectively. Thus, a vector [*x*_1_-*x*_0_, *y*_1_-*y*_0_] represents the orientation of the afterfeather. We found that the left-handed afterfeather descends with counterclockwise rotation, while the right-handed one falls with clockwise rotation. The same motion can be replicated using a vertical wind tunnel, providing a stable flow field of the same velocity as the descent speed. Details of the wind tunnel can be found in note S2. Inside the wind tunnel, the structure’s weight is balanced by the aerodynamic forces, resulting in a self-sustained motion in midair. Figure 1C shows the historical path of the rotation observed from a top-view angle. As before, distinct rotary directions are presented by afterfeathers with different handedness. This natural feather model can combine both automation mechanism and porosity-induced airborne stability as introduced previously in two different categories in the context of bioinspired passive flight. Details of rotation and stability inside the wind field can be seen from movie S1.

Here, we adopt a strategy to harness the chirality of afterfeathers to realize light-controlled rotational flight, with a design schematically shown in Fig. 1D. A photomechanical soft strip actuator with distinct deformation modes, namely, bending and coiling (*34*, *35*), is used to build the connection between two afterfeathers with the same or opposite handedness. The strip actuator is based on LCN, which is ultralight (1.6 mg/mm^3^, 0.13 mg inside the structure) and actuated quickly upon light excitation with a suitable wavelength. The sample preparation is described in note S3. With this soft actuator, the feather assembly herein has the manual control of the degree of freedom in system’s handedness by activating the twisting or structural opening angle using the bending mode. These two actuation modes switch the structural handedness and can tune the rotation speed and altitude in the midair, which will be elaborated in the following sections.

### Twirling in feather assemblies

**Fig. 2.**
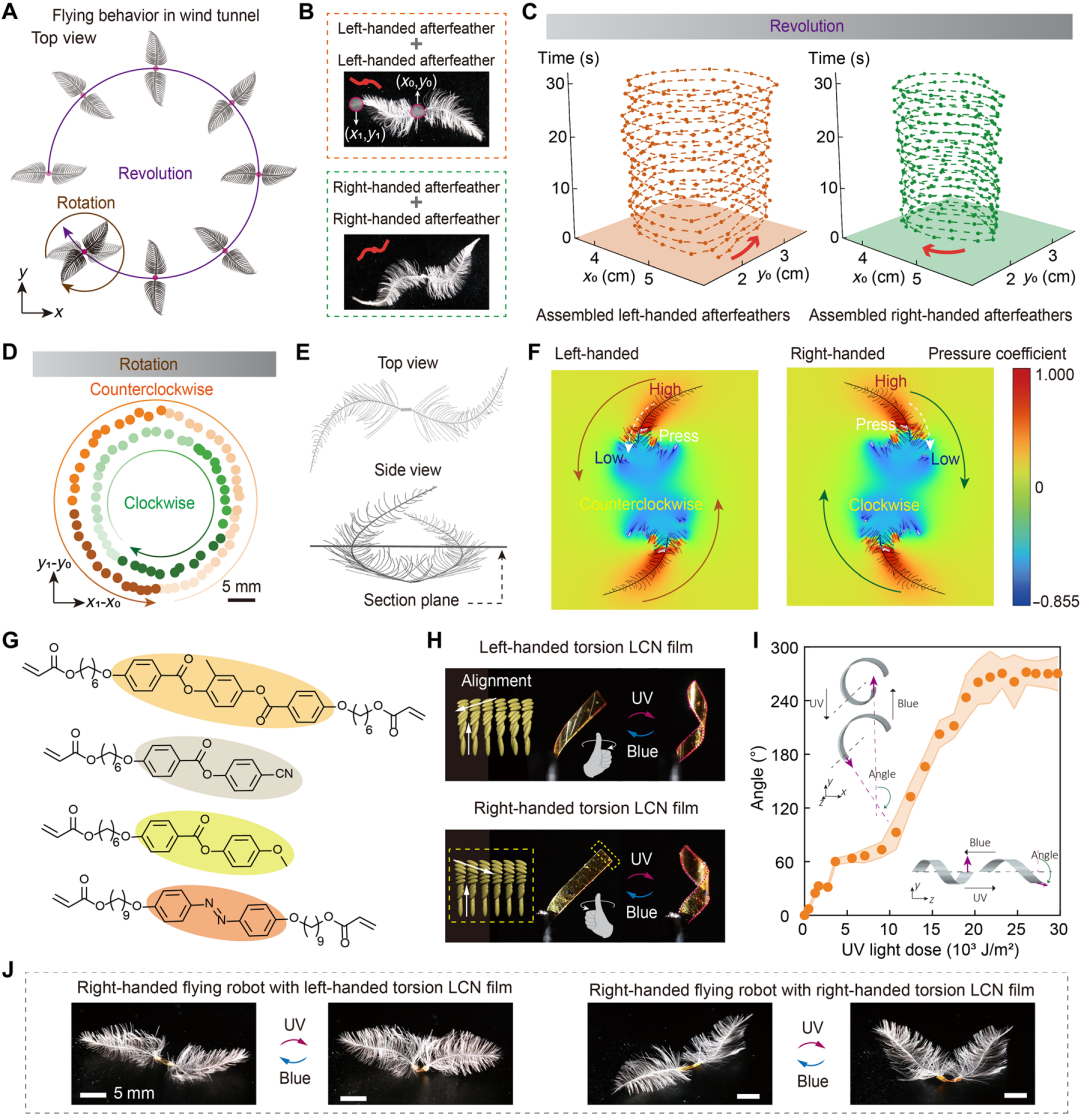
Handedness of chiral afterfeather assemblies and LCN film torsion. (**A**) Flight behaviors of afterfeather assemblies in the wind tunnel: revolution and rotation. (**B**) Assembly types of chiral afterfeathers. (**C**) Gliding revolution of afterfeather assemblies in the wind tunnel. The revolution is characterized by the trajectory of the feather assemblies’ centroid. (**D**) Gliding rotation of afterfeather assemblies in the wind tunnel. The rotation is characterized by the relative trajectory between its centroid and tip. (**E**) Top and side views of the simulated model for afterfeather assemblies. (**F**) Wind tunnel simulation results of afterfeather assemblies at the section plane, showing counterclockwise (left-handed) and clockwise (right-handed) rotational flight. (**G**) The molecular formula of LCN. (**H**) The liquid crystal alignment in LCN films and their UV-induced left-handed and right-handed torsion deformation. (**I**) Relationship between torsion angle of LCN film and UV light dose. Data are shown as mean ± SD (*n* = 3 independent samples). The insets show schematic drawings indicating the torsion angle during the coiling-uncoiling deformation of the LCN. (**J**) Two types of gliding feather-actuator assemblies: right-handed afterfeathers with left-handed torsion LCN film and right-handed afterfeathers with right-handed torsion LCN film.

A mechanical connection by directly gluing two pieces of afterfeather forms the twirling feather assembly. The midair behavior exhibits two types of movement, i.e., rotation and revolution, as schematically shown in Fig. 2A. Rotation refers to the body’s rotary movement around the assembly axis, which is located at the geometrical center of the assembly. Revolution refers to an orbital motion along a circular trajectory when considering the gliding-induced displacement of the mass center (*x*_0_, *y*_0_). As shown before, each afterfeather has inherent chirality. Therefore, the handedness of midair rotary and revolutionary movements is expected to be determined by the inherent chirality of the afterfeather being integrated. Figure 2B shows the examples of the connection between two left-handed afterfeathers and two right-handed ones. The third connection possibility is left-handed and right-handed afterfeathers, which exhibits an unstable gliding state inside the wind field owing to its asymmetric structure (see movie S2 and fig. S2). By tracking the position of the center of the mass, Fig. 2C presents a directional revolution in the wind tunnel: left-left assembly exhibits a counterclockwise revolution, while right-right assembly shows a clockwise revolution. Details of the revolutionary trajectory are given in figs. S3 and S4, which shows 2.9 to 3.7 rad/s speed, 1.68- to 2.17-s period, and 1.4- to 2.3-cm orbital radius.

Recording the movement within the centroid coordinate system reveals the rotary motion, as shown in Fig. 2D. Similarly, left-left assembly shows a counterclockwise rotation, while right-right assembly exhibits the rotation of reversed direction. To understand the aerodynamics, we perform computational fluid dynamics (CFD) simulation to analyze the pressure field distribution by mapping the pressure coefficient *C*_p_ around a manually sketched feather-mimic structure fixed in a steady wind flow. The pressure coefficient is described in Materials and Methods. The bionic structure, shown in Fig. 2E, consists of 46 fine hairy filaments arranged along each chiral backbone. The length of the hairy attachment gradually decreases along the backbone from the root to the tip, following the configuration of a natural afterfeather. Details of the time evolution of aerodynamic torque acting on the afterfeather-LCN assembly and the simulated angular velocity are provided in note S4. Movie S11 also shows the temporal evolution of the pressure field. The CFD results shown in Fig. 2F reveal that along the growth direction of the feathers, *C*_p_ is higher near the outer sides of the filaments and lower near the inner sides. This pressure gradient drives air to flow from high-pressure regions toward low-pressure regions (*36*). The handedness of the assembly determines the orientation of this flow, thereby inducing different handedness in rotary motion; for instance, a left-left assembly rotates counterclockwise, while a right-right assembly rotates clockwise.

We then try to integrate two feathers with a soft strip actuator and use light to photomechanically tune the rotation in the midair. The actuator is photopolymerized from an LCN mixture, of which the chemical structures are given in Fig. 2G. This specific type of LCN can deform reversibly upon ultraviolet (UV) and visible light illumination under isothermal conditions. This photomechanical deformation shows no degradation over 100 actuation cycles (fig. S5). This mechanism is induced by the photoisomerization of azobenzene molecules at the crosslinking position within the polymeric chains, the properties of which have been well studied within the LC polymer community (*37*, *38*). In our cases, the LCN is fabricated within a 20-μm-thick splayed-aligned cell, UV polymerized, and cut into strips with 1-mm width. Because of the small thickness, the actuator has low weight, i.e., 0.03 mg/mm. We adopted two specific cutting angles that are 45° and −45° between the strip and the LC director, which result in coiling deformation with left- and right-handedness. The schematic drawing of the LC alignment at the cross section and the two handedness of photo-induced deformation upon subsequent UV and blue light irradiation are shown in Fig. 2H. The coiling angle is indicated by the insets of Fig. 2I, which can be finely tuned through exposure to UV of different doses (Fig. 2I). A 180° change of coiling angle means a flipping over of the LCN strip end or any lightweight objects attached to it. If the object has certain chirality, then the LCN deformation results in a change of handedness of the assembly. Figure 2J shows the photographs of the LCN-feather assembly before and after the photoactuation. A left-handed torsion LCN can switch a right-right assembly into a right-left assembly, while a right-handed torsion LCN strip can change a right-right assembly into a left-right one.

### Midair light control of chirality

**Fig. 3.**
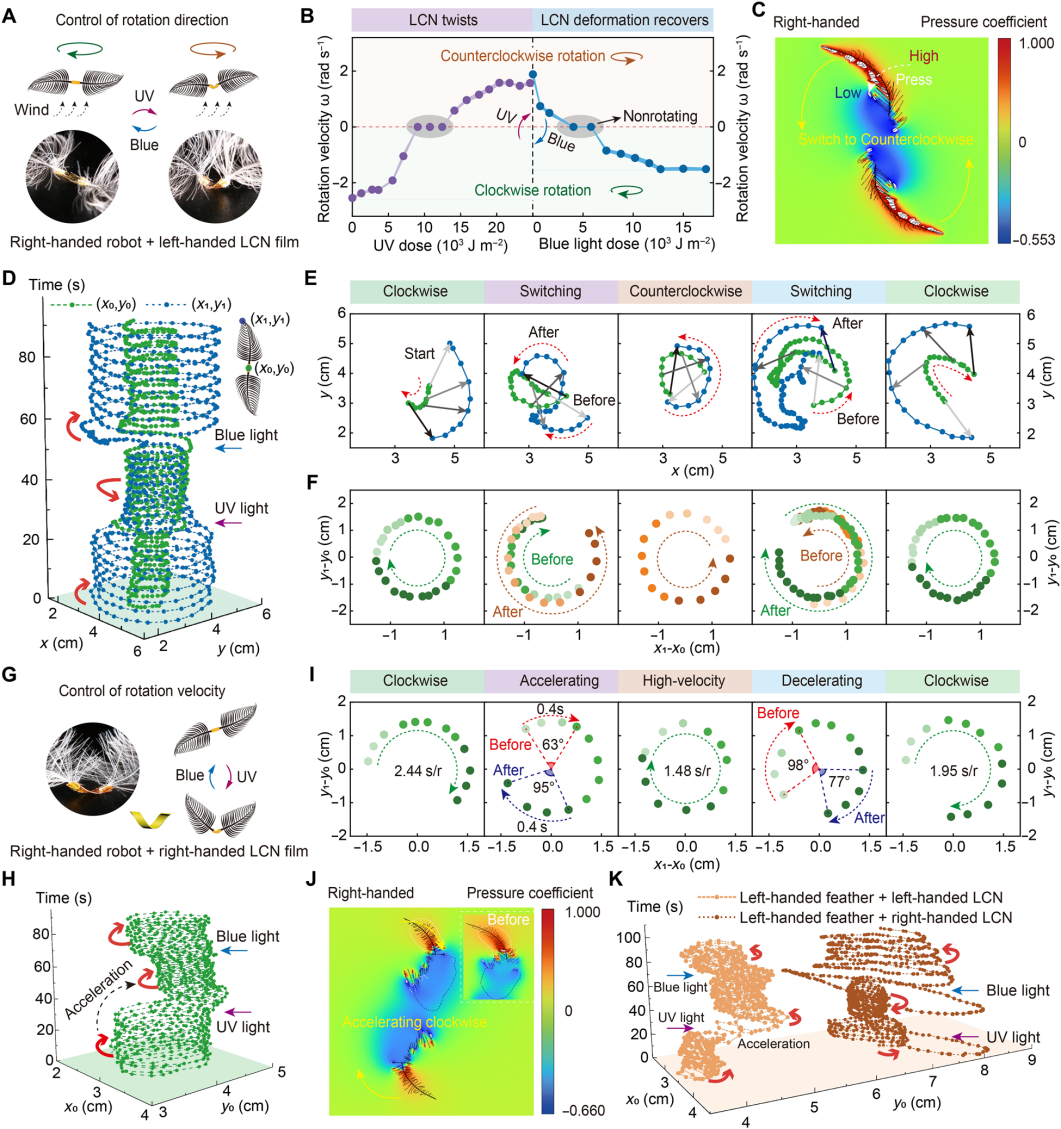
Light-induced rotational regulation. (**A**) Control of rotation direction for right-handed afterfeathers via left-handed LCN film. UV-induced torsional deformation of LCN triggers rotation direction reversal, while blue light restores the original rotation. (**B**) Rotation velocity versus UV/blue light dose in LCN twisting-recovery cycles. (**C**) Simulated counterclockwise rotation switching in right-handed afterfeather via twisted left-handed LCN film. (**D**) The gliding trajectory of the feather-actuator assembly’s centroid and tip during the process of UV-induced rotation direction reversal and blue light–induced recovery. (**E**) Trajectory variations of centroid and tip positions across characteristic stages. (**F**) Rotational variations across characteristic stages. (**G**) Control of rotation velocity for right-handed afterfeather assembly via right-handed LCN film. UV-induced torsional LCN deformation accelerates rotation, while blue light restores the rotation velocity. (**H**) The gliding trajectory of the feather-actuator assembly’s centroid during the process of UV-induced rotation acceleration and blue light–induced recovery. (**I**) Rotational variations across characteristic stages. (**J**) Simulated rotation acceleration in right-handed afterfeather assembly via twisted right-handed LCN film. (**K**) Light-fueled rotational regulation of the left-handed afterfeather assemblies.

Here, we implement two right-handed afterfeathers connected by an LCN actuator with left-handed torsion in photodeformation. As a connector, the LCN strip exhibits a nearly flat shape originally and can reversibly twist the right-hand chiral afterfeathers on both sides toward a left-handed direction upon light irradiation (Fig. 3A and movie S3), while the configuration of the central actuator has a negligible influence on the aerodynamic behavior of the entire cluster (fig. S6). After placing the assembly inside the wind tunnel with steady wind flow, rotation speed (ω) is recorded upon different UV/blue light dose as explicated in Fig. 3B. Originally, it shows a 2.4 rad/s rotation clockwise. Being illuminated with around 10^4^ J/m^2^ doses of UV, the rotation ceases and then switches to the counterclockwise direction upon further elevated illumination intensity. Turning off the UV and illuminating the assembly with blue light result in a reversal of rotary direction. The counterclockwise rotation quickly stops after receiving a dose of 4 × 10^3^ J/m^2^ doses of blue light and then returns to the clockwise direction upon prolonged illumination. Figure 3C shows the CFD result of the *C*_p_ around the deformed assembly, which is distinct from the one before photoactuation as explicated earlier in Fig. 2F (right). Before actuation, both afterfeathers stay flatly on the horizontal plane, resulting in high-pressure and low-pressure zones on both sides of the shaft (Fig. 2F). After deformation, both afterfeathers are tilted upward inside the wind flow, and this modifies the pressure distribution: High-pressure zones exhibit around the outer part of the tip portion of the afterfeather, while low-pressure regions are confined around the internal side of the basement. To reduce the meshing complexity and computational cost associated with resolving the 3D feather hairy geometry, we used a simplified 2D porous plate model in the CFD simulations. A porosity of 0.51, measured from natural samples (note S1), was prescribed to capture the aerodynamic response of the feather assembly and to estimate the resulting aerodynamic torque. The simulations reveal a reversal in the net aerodynamic torque at a critical interfeather angle and demonstrate that the torque can be continuously tuned through torsional deformation. Details of the model and the torque-reversal analysis are provided in note S5.

On the basis of the fact that LCN actuation can be programmed through light input and reversibly tuned by switching between UV and visible light, one would expect a precise midair control of the rotary motion. Movie S4 shows the light-induced handedness switching of the same feather assembly, and Fig. 3D shows the recording of the horizontal trajectory. Note that (*x*_0_, *y*_0_) indicates the revolution of the center of mass, and the sample orientation (for rotation) is presented by [*x*_1_-*x*_0_, *y*_1_-*y*_0_]. The thin waist of the spiral trajectory indicates the deformation of the assembly upon photomechanical actuation that reduces the expanded width on the *xy* plane. The whole trajectory can be divided into five phases, i.e., original clockwise motion, UV switching, counterclockwise, back switching, and return to clockwise rotation. The typical gliding behaviors in each phase are presented by mass/tip position and orientational vector in Fig. 3 (E and F). They clearly show that a stabilized configuration corresponds to a stable rotation/revolution in a clockwise or counterclockwise direction; handedness switching is witnessed by the crossing between vectors in Fig. 3E and the reversion of rotation directions in Fig. 3F.

Connecting two right-handed afterfeathers with one right-handed LCN actuator results in a light-controlled rotation speed within the same form of handedness. Figure 3G and movie S5 show that an LCN strip can reversibly twist the right-handed afterfeathers toward a right-handed direction upon light irradiation. Coiling the LCN with the same handedness as the one of the afterfeather enhances the power of chirality in the assembly, as shown in Fig. 3G. Performing the same experiment inside the wind tunnel, different rotation velocities are recorded in Fig. 3I, i.e., the period to complete a clockwise rotation (2.5 s) can be reduced to 1.5 s and then prolonged to around 2 s. An accelerating/decelerating phase is indicated by the enhanced/reduced rotary angle across an identical time span (0.4 s). The details of the trajectory about the photo-induced acceleration and deceleration upon UV and blue light excitations are shown in Fig. 3H, in which the thin waist indicates an acceleration of rotation, in this case, of the same handedness. The CFD simulation shows the isometric map of the assembly, revealing that an enlarged low-pressure area emerges right after the photomechanical actuation (Fig. 3J). The enhanced pressure force elevates the air flow and thus accelerates the rotation without changing the handedness. In a brief conclusion, the connection between chiral afterfeathers and a handed LCN actuator can introduce different types of midair control of rotation/revolution velocity and light switch between left- and right-handedness in motion (Fig. 3K). The rotational variations across characteristic stages can be found in figs. S7 and S8.

### Light-controlled elevation and descent

**Fig. 4.**
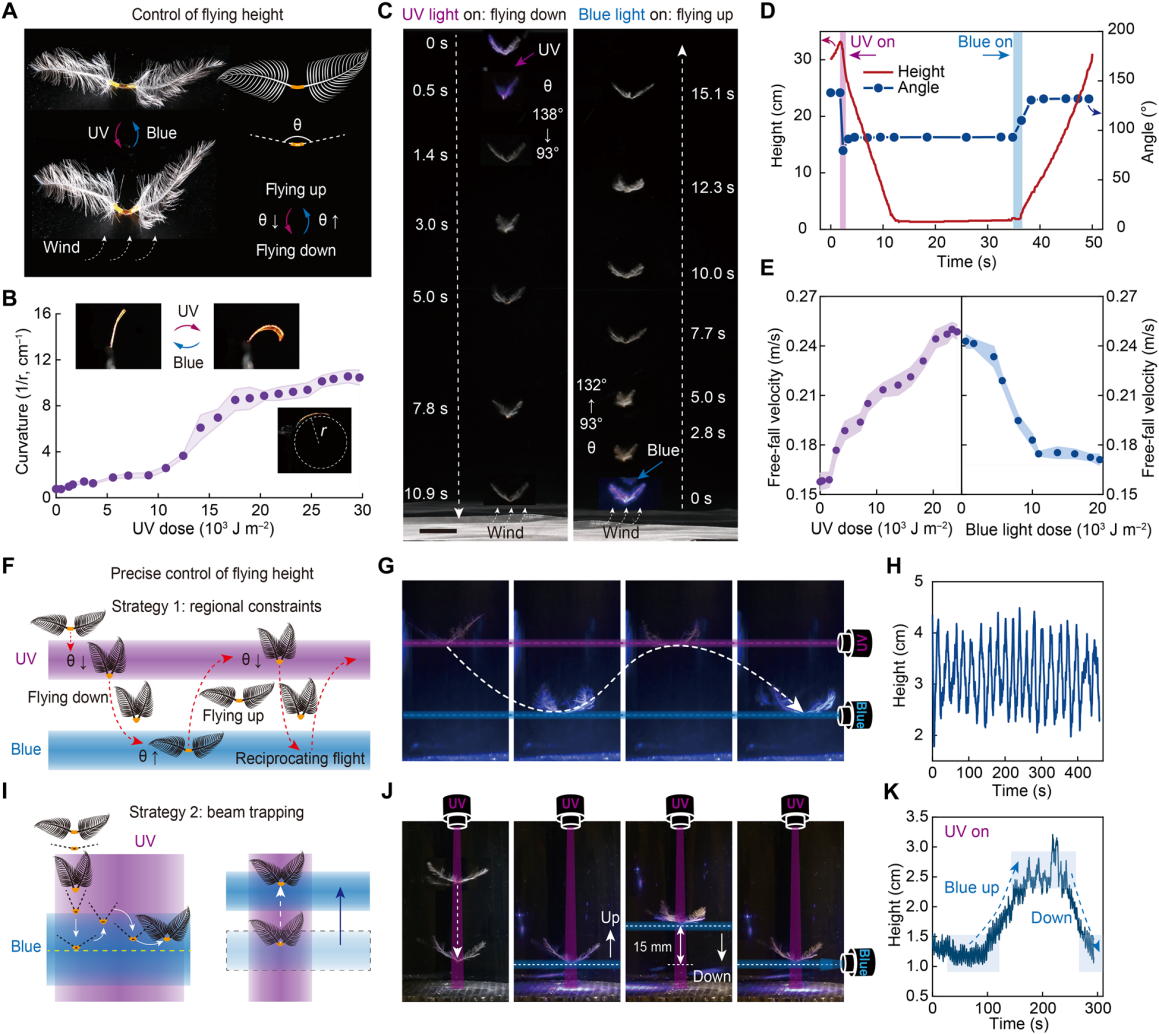
Light-fueled flight height regulation of flyer via LCN bending deformation. (**A**) Flight height regulation of the flyer via wing angle control using bent LCN film. (**B**) Relationship between curvature of LCN film and UV dose. Data are shown as mean ± SD (*n* = 4 independent samples). (**C**) UV-induced LCN bending reduces wing angle, causing the robot to fly down; conversely, blue light triggers upward flight. (**D**) Variations in flight height and wing angle of the flyer during the process in (C). (**E**) Free-fall velocities versus UV dose (left axis) and blue light dose (right axis). (**F**) Principle of constraining the flyer to a defined flight height region. (**G**) The flyer flight reciprocates between UV and blue light beam zones. (**H**) Time-dependent gliding height variations in reciprocating flight. (**I**) Principle of trapping the flyer at a defined flight height. The flyer is trapped in the upper half of the blue beam crossing the UV beam. (**J**) The flyer is captured by the blue light beam, lifted upward by 15 mm, and then precisely controlled to descend back to the original capture height. (**K**) Time-dependent gliding height variations in beam trapping flight.

Usually, the stable flow provided by the wind tunnel is inherent with a certain velocity gradient, i.e., the flow speed gradually increases with distance further away from the output. The measurement of the velocity gradient of the wind tunnel is explained in note S2. This gradient allows an altitude modulation if one can alter the effective area of the assembly. Here, we use a bending mode LCN actuator to connect two afterfeathers with the same handedness. The photomechanical bending of LCN can bring a light-tuned opening and closure of the feather assembly, therefore modulating the effective surface. The photographs and schematic drawing of the photomechanical deformation of the feather assembly are shown in Fig. 4A and movie S6. In this case, the LCN is fabricated with the same chemical composition (Fig. 2G), thickness, and alignment as the ones used in rotational control. We cut the LCN strip along the LC direction, which gives a bending deformation without coiling. The curvature variation upon different UV exposure is shown in Fig. 4B. A flat LCN strip can morph into a curved shape over 10 cm^−1^ in curvature, meaning a tightly closed ring geometry with around 1 mm radius after the photomechanical bending.

Figure 4C shows the snapshots of light-induced altitude change inside the wind tunnel (see movie S7). At the beginning, the feather-actuator assembly stays at a height of 32 cm above the wind output (the plate of honeycomb, referred to as the ground). By tuning the assembly’s opening angle from 138° to 93° with an UV shot, the assembly descends to near the ground, at which the feather experiences higher air drag due to the elevated flow velocity. A blue light illumination triggers backward deformation, opening the configuration and elevating the assembly to a larger height. The altitude variation together with the change of opening angle is recorded in Fig. 4D, which shows a correlation between the irradiation moment (UV or blue light), and the change of the height and configuration. As the opening angle can be quantified by irradiation dose, the feather assembly has programmability in a descent velocity through proper exposure between UV and blue light. Figure 4E quantifies this relationship between descent velocity and light dose by measuring free-fall velocity in still air. Accordingly, the flying up and down height of assembly can also be tuned by light-controlled angle change, as shown in figs. S9 and S10.

The precise control of midair objects conventionally requires a closed-loop system, where a camera is used to monitor the position and a controller is used to change the irradiation dose to ensure the flyer stays at a predetermined altitude. Here, we introduce two methods to obtain altitude control that fully relies on LCN’s stimuli responsiveness without any electronic control. Figure 4F shows the first strategy based on regional light constraints. A UV light beam propagates at a higher altitude, while a blue light beam propagates at a lower altitude. A feather assembly experiences rebounding between two light beams while being sandwiched (Fig. 4G and movie S8). With a big opening angle, the assembly flies upward to touch the line of the UV beam; once exposed to UV light, it closes the configuration and starts to descend. Once it descends to the lower line of blue illumination, it opens the configuration and elevates toward the UV line. Encountering the UV zone as the beginning, the assembly repeats the same cycle by showing oscillation between two light beams. Therefore, it has been dynamically constrained within the two-beam space. The confinement is indicated by the oscillating trajectory in Fig. 4H, which never exceeds the light beam boundaries. The second strategy is based on one beam trapping mechanism, as schematically shown in Fig. 4I. Instead of propagating horizontally, the UV light illuminates the whole area along the entire altitude. A horizontal blue beam placed at the bottom serves as the trapper to constrain the movement of the assembly. In this case, the flyer tends to close in shape to drop inside the tunnel at whichever altitude, due to the global UV illumination. Once the assembly touches the blue beam, at which the intensity of blue illumination is stronger than that of the UV, it opens and moves upward. After elevating away from the blue region, it encounters the pure UV region and starts to close and descend, repeating the same cycle. The assembly is dynamically confined at the altitude just above the level of the blue beam. A change of blue beam altitude can manually adjust the height of the assembly, as shown in Fig. 4 (J and K).

It is worth noting that, under different wind tunnel flow velocities, manual irradiation with appropriate doses of UV/visible light enables the fixation of the flyer height within the wind tunnel. In this case, photomechanical actuation is used to reconfigure the flyer geometry such that the weight and drag forces are balanced. At this aerodynamic equilibrium, the spinning rate directly reflects the airflow velocity (fig. S13). Details of drag coefficient (*C*_D_) calculation are provided in Materials and Methods.

### Individual control and massive collection

**Fig. 5.**
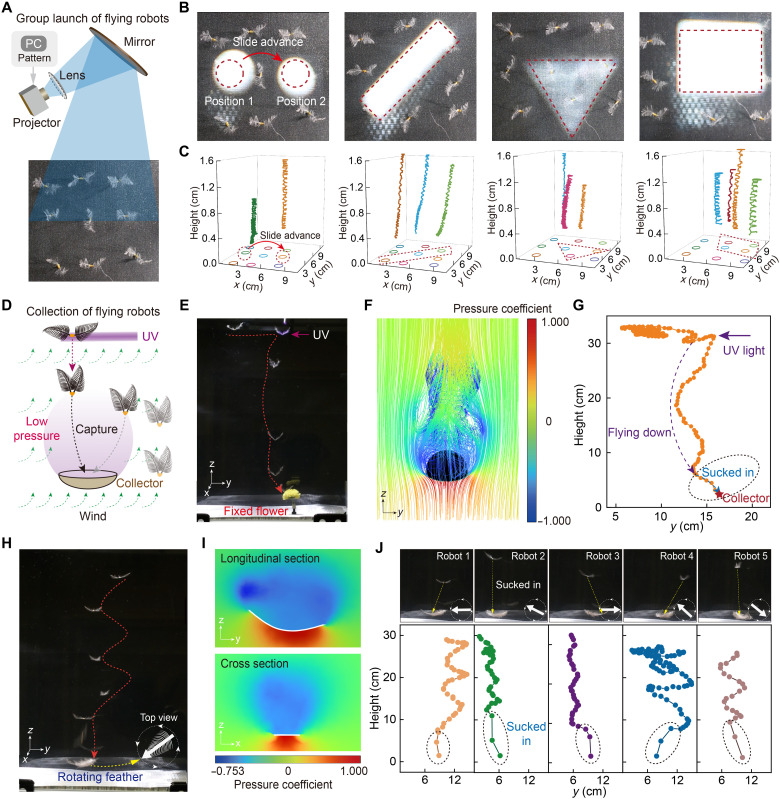
Programmable launch and collection of the flyers. (**A**) Schematic diagram of the system setup for controllable group launch of flyers. (**B**) Arbitrary shape projection patterns for triggering programmable group launch of flyers. (**C**) The corresponding flight trajectory of the launched flyers. (**D**) Collection principle of the flyers based on the low-pressure effect above the collector. (**E**) The flyer is UV-induced to fly down and then captured by a stationary flower. (**F**) Flow field simulation results around the flower in the wind tunnel. (**G**) Variations in flight height of the flyer during the process in (E). (**H**) Capture process of flyers using a dynamically rotating feather collector. (**I**) Flow field simulations of feather longitudinal and cross-sectional profiles in the wind tunnel. (**J**) Five flyers were captured by the dynamic feather collector at varying rotation angles.

Light actuation provides merits in individual control of multiple objects due to the high precision in spatial intensity distribution. Here, we demonstrate the possibility of individual control among multiple feather-actuator assemblies within the same wind flow. Figure 5A shows the principle, where an optical projector is used to project a pattern of white light source after being collimated and reflected onto a horizontal plane inside a vertical wind tunnel. At the wind output, there stand nine feather-actuator assemblies, each of which actuator is made of bending LCN for altitude control. All the assemblies are preilluminated with 2 × 10^4^ J/m^2^ UV dose, to maintain a closed configuration and stay still inside the wind flow whose velocity is lower than the assembly’s descent speed. Projection of light patterns with different geometries, i.e., round shape, bar, triangle, and rectangle, activates different assemblies at different positions (Fig. 5B and movie S9). Upon light excitation, the assemblies open the configuration and take off inside the wind flow. The time-dependent positions of the centroid clearly indicate the light selected take-off among assemblies. 3D trajectories among all assemblies for each light pattern are depicted in Fig. 5C.

To efficiently harvest the wind-dispersed flyers, we introduce two methods of midair collection. The first one is based on a static collector, as schematically shown in Fig. 5D. An impermeable object placed inside the tunnel redirects the wind flow downstream and generates a region of low pressure and low airflow. Such a region leads to the collection of any flying object approaching nearby. Figure 5E shows the snapshots of a feather-actuator assembly performing a self-collection action taken by a natural dandelion flower (2 cm in diameter, fixed at the wind output). After exposure to UV at high altitude, the assembly starts to descend and, after deviating from its original vertical path, suddenly turns toward the flower and enters its capturing region at the bottom. The CFD-simulated distribution of the static pressure coefficient is shown in Fig. 5F, indicating the region of collecting above the flower. Tracking of midair position in Fig. 5G shows a random movement inside the wind flow that is disturbed by the collector. A small spacing between two positional points (0.13-s interval) reveals a slow motion during the wind-assisted gliding and light-induced descent, while a suddenly enhanced spacing near the collectors indicates a rapid attractive motion.

The second way of collection is implemented using a natural feather as a dynamically moving collector. Figure 5H shows the snapshots of an actuator assembly being received by a feather. A feather as shown earlier in Fig. 1A has an asymmetric architecture that allows it to sustain in the air and glide around the bottom of the wind tunnel. In this case, the feather acts as a collector, capturing nearby midair objects that drift downward and then abruptly veer toward it (see details in movie S10). The CFD-simulated pressure zones in longitudinal and cross sections indicate the frontier of the collective area (Fig. 5I). Five different feather assemblies are released into the wind tunnel from the top, and all of them are successfully captured by the collector. The photographs of the flyer and the corresponding height tracking are shown in Fig. 5J. In this case, the collection is performed at varying locations, as indicated by the sudden movement trajectories near the collector.

## DISCUSSION

This study addresses the issue of midair control of altitude and rotation speed in a passive flyer. However, these two kinds of controllability are achieved using two different LCN actuators, which are handed coiling LCN for rotation control and bending LCN for altitude modulation. When considering the 3D position control in the future, one may combine both control strategies within one flyer structure, namely, an integrated assembly consisting of two LCN strips with distinct deformation modes and three afterfeathers. In such a case, a bending of the configuration gives rise to an altitude change in the *Z* direction, while a cessation and revival of revolutionary gliding with a large orbital radius bring a benefit to reaching any position on the *xy* plane. The basic design concept behind is the enhancement of the degree of freedom for optical adjustment in midair motion through simply using more LCN actuators. It should be noted that, because of natural variability, the rotational speed varies between samples (fig. S12). However, the underlying principle, that deformation of the LCN governs the aerodynamic behavior of the structure, remains consistent across all samples.

Vortices play a substantial role in aerodynamics. An axi-symmetric vortex is associated with stable low pressure and airborne stability for the seeds of the dandelion (*23*). Asymmetric vortices provide an air pressure gradient that dictates the rotatory and revolutionary direction and velocity. These vortices can also be used to collect feathers from the air. The reconfiguration of geometry in this study, namely, the change of chirality, has been achieved using LCN actuation. Nevertheless, vortex-induced clustering may bring out diverse configuration. For instance, two centrally symmetric flyers connect each other to form a mirror-symmetric cluster; three centrally symmetric flyers cluster into a chiral glider. The random clustering between flyers due to vortex interaction could introduce novel midair behaviors.

The experiments in this study were performed inside a controlled wind tunnel. In nonideal conditions, airflow often includes cross-gusts, updrafts, descent flow, etc. Because of its ultralight weight (∼1 mg) and very low descent velocity (around 0.2 m/s), the feather robot can effectively follow the surrounding airflow, thereby serving as a tool for visualizing ambient atmospheric flow through observed changes in position and spinning rate (fig. S11). From a robotics perspective, when exposed to side winds, the stimuli-responsive material allows the flyer to adapt autonomously. For example, within an illuminated region, the flyer can take off (as demonstrated in Fig. 5B) and achieve side wind–assisted translocation. Under steady updraft conditions, the spinning rate at aerodynamic equilibrium quantitatively reflects the velocity of gentle airflow as shown in fig. S13.

There are two main limitations in the projection-based control strategy. First, the light intensity decreases as the illuminated area expands. In other words, a higher-power projector is required as the number of individually controlled flyers increases. While multibeam steering can provide laser excitation with a more spatially confined intensity field, it also increases the complexity of the control system. Second, low downstream airflow can induce unintended attachment between two flyers when they approach within a certain distance. The results shown in fig. S14 quantify the critical horizontal overlap distance to be 6 to 8 mm, beyond which two flyers are likely to adhere to each other during midair rotation.

The active MAVs often rely on electric motors (*39*), piezoelectric (*40*), or dielectric actuators (*41*) for the flight. In that system, energy efficiency is the most crucial issue. Therefore, high-powered actuators/motors are implemented to produce sufficient air thrust to sustain the movement in the midair. They hence face challenges in untethered control and miniaturization. Wind-dispersed seeds in nature, such as dandelions, maple seeds, and poplar tree seeds, offer inspiring examples for MAV’s operation and maneuvering using passive flight modes combined with astonishing energy efficiency. The bioinspiration for human-made flyers is that the manual input energy is only to steer the flight direction or switch between different behaviors, while a major part of the energy consumption (balancing the gravitational force) is adjusted by natural winds. As novel examples are pioneered in very recent years (*14*–*16*, *24*, *42*), this study provides access to altitude control (*Z*) and azimuthal orientation (φ), within full control requirement (five degrees of freedom: *X*, *Y*, *Z*, θ, and φ). We expect an increase of attention and more research in this nascent field.

In conclusion, we report a robotic assembly by integrating an LCN strip actuator with two chiral afterfeathers taken from a Guinea fowl. The azobenzene-functionalized groups within the LCN polymeric network enable actuators’ shape reconfiguration upon UV and blue light irradiation, during which the coiling angle and bending angle can be precisely programmed using different light doses. The handedness of the assembly is determined by the chirality of the integrated feather and can be modified by photomechanical deformation of the LCN strip. We achieved light-controlled rotational speed between −2 and 2 rad/s, switch of rotary and revolutionary handedness in midair, and acceleration and deceleration of rotation/revolution. CFD simulations reveal the details of air pressure field around the flyer, the gradient of which dictates the rotation/revolution direction. The LCN actuation triggers a redistribution of high-low pressure zones that switches the motion chirality. The bending mode of the LCN actuator induces opening and closure of the assembly, resulting in a light-controlled elevation and descent inside the wind gradient over 30-cm travel. The altitude is precisely controlled via dynamic light–field confinement, enabled by the self-regulation of the responsive material, which establishes an intrinsic feedback mechanism (*43*) between the flyer and the optical field, without the need for any electronic feedback. Besides, individual control among nine feather-actuator assemblies for performing take-off action is achieved by using projected light patterns. Dynamic and static collectors are used to harvest feather assemblies in the midair. The key results of the present research, such as the combination of chiral natural feathers with handedness of the actuator and the associated light-triggerable controls, can serve as a comprehensive toolbox for design and control of future micro robots in the air. We hope our study can also provide a simple but effective experimental platform to study micro-scale flying robotic devices and their interactions with aerodynamics.

## MATERIALS AND METHODS

### Materials

4-Methoxybenzoic acid 4-(6-acryloyloxyhexyloxy)phenyl ester (ST03866), 4-[4-(6-Acryloxyhex-1-yl)oxyphenyl]carboxybenzonitrile (ST02670), 1,4-bis-[4-(6-acryloyloxyhexyloxy)benzoyloxy]-2-methylbenzene (ST00975), and 4,4′-bis[9-(acryloyloxy)nonyloxy]azobenzene (ST04181) were purchased from SYNTHON Chemicals. Polyvinyl alcohol, bis(2,4,6-trimethylbenzoyl)-phenylphosphine oxide (819) was obtained from Sigma-Aldrich. Polyimide (used as liquid crystal aligning agent) was purchased from SJLLASER (HONGKONG) Technology Limited. All chemicals were used as received. Feathers samples are guinea fowl feathers (brown color) purchased from cchobby.com.

### Fabrication of feather-actuator assembly

The obtained LCN film was first cut into strips with dimensions of 4 mm by 1 mm by 20 μm. Depending on the cutting direction, three types of deformation modes could be achieved: bending, left-handed torsion, and right-handed torsion. To fabricate the feather-actuator assembly, two afterfeathers were attached to both ends of the LCN strip using UV-curable adhesive, as shown in fig. S15. By combining the three types of LCN strips with either left or right afterfeathers, a total of six different assemblies were obtained (see fig. S16). The total weight of the assembly is about 1.0 mg in the experiments.

### Chiral afterfeather test

To eliminate the influence of external airflow and environmental disturbances, we constructed a transparent tunnel. The left- or right-handed afterfeather was then released to undergo free fall inside the tunnel. The falling process was recorded using a camera with a frame rate of 120 fps, and the falling velocity, as well as the rotation direction, was subsequently analyzed. All free-fall experiments were performed in this tunnel.

### The rotation control experiment of the feather-actuator assembly

The assemblies were first placed at the bottom of the wind tunnel. The wind tunnel was then activated, and the airflow speed was adjusted to allow the assemblies to ascend smoothly and stably glide. Subsequently, a laser beam was used to irradiate the robot, inducing shape changes such as left- or right-handed torsion deformation. This resulted in a change in the assemblies’ flight posture or motion. The entire process was captured as video. Last, the rotational direction and speed of the assemblies were extracted from the video files and analyzed using motion analysis software Tracker.

### Light-controlled descent and ascent experiment

The flyer was first placed at the bottom of the wind tunnel and lifted to a stable position near the top by adjusting the wind speed. A 385-nm UV laser was then illuminated from above to induce bending, causing the flyer to descend. Upon reaching the bottom, a 460-nm blue laser was irradiated onto the robot to reverse the deformation, enabling it to ascend again. Through this light-controlled process, the flyer completed a descent-ascent motion.

### Regional constraining of flyer experiment

A UV laser (385 nm) and a blue laser (460 nm) were positioned laterally outside the wind tunnel, aimed at fixed heights near the top and bottom, respectively. After initiating stable flight, the flyer was exposed to alternating light zones: It bent and descended upon entering the UV region and recovered to ascend when reaching the blue light zone. This configuration allowed the flyer to oscillate vertically between two light-controlled deformation zones.

### Beam-induced trapping of flyer experiment

In this setup, the UV laser (385 nm) was directed vertically from the top of the wind tunnel, while the blue laser (460 nm) remained on the lateral side. After the flyer was stabilized at the top, both lasers were activated. The flyer bent and fell under UV exposure and then unbent and rose slightly when exposed to side irradiated blue light. By adjusting the vertical position of the blue laser, the flyer’s hovering height could be dynamically modulated.

### Selective control of flyer array experiment

To realize spatial control of a 3 × 3 flyer array, a commercial projector was modified to project arbitrary light patterns. Specifically, the original lens was removed and replaced with a custom lens system to focus the output beam to the bottom of the wind tunnel. Pattern control was achieved by projecting predesigned slides (e.g., Microsoft PowerPoint content), allowing dynamic adjustment of illuminated regions. In the experiment, nine flyers with bending deformation were placed at the bottom of the wind tunnel. Before the experiment, all flyers were uniformly irradiated with 385-nm UV light to induce a bent (inactive) state, keeping them at the bottom under airflow. Upon projection of a selected light pattern using the modified projector, robots located within the illuminated region received white light, triggering shape recovery and takeoff. By varying the projected light pattern, such as round, bar, triangle, or rectangle, specific subsets of the array could be selectively activated and made to fly.

### Static collection experiment

A flower was placed at the bottom of the wind tunnel. The wind tunnel was turned on to establish a stable upward airflow. A flyer was then introduced into the tunnel. When the flyer happened to fly near the top of the flower, it was captured and adhered to the flower surface. The entire process was recorded, and the landing trajectories of the robots were extracted for analysis.

### Dynamic collection experiment

A big feather was first placed at the bottom of the wind tunnel. After turning on the wind tunnel, the feather maintained stable flight near the lower region. Flyers were then introduced into the tunnel one by one. It was observed that each flyer was gradually drawn toward and captured by the large feather. The entire process was recorded, and the trajectories of the robots during the capture process were extracted and plotted for further analysis.

### CFD simulation

Numerical simulations were carried out using ANSYS Fluent 2023R2, with all parameters detailed in notes S4 and S5. The pressure coefficient $C_{p}=\frac{p-p_{\text{ref}}}{\frac{1}{2}\rho V_{\text{ref}}^{2}}$, where *p* is the local static pressure, $p_{\text{ref}}$ is the reference static pressure, ρ is the fluid density, and $V_{\text{ref}}$ is the reference velocity. A uniform free-stream velocity was prescribed at the velocity inlet, while a uniform free-stream pressure was imposed at the pressure outlet. The inlet plane was used as the reference plane for evaluating the pressure coefficient. The fluid domain was a cylindrical volume with a diameter of 100 mm and a height of 80 mm.

### Drag coefficient calculation

The drag coefficient (*C*_D_) of (mass = 0.80 mg) was evaluated via drop tests conducted from a height of 0.5 m under quiescent air conditions. Each test was repeated three times, and the mean descent speed was determined from video recordings captured using a Canon EOS 60D camera. The terminal velocity was extracted using Tracker software. The drag coefficient *C*_D_ was calculated from the force balance at terminal velocity, where gravitational force equals aerodynamic drag: $\mathrm{mg}=\frac{1}{2}\rho_{\text{air}}v_{t}^{2}{\mathrm{AC}}_{D}$, where *m* is the mass of the flyer (0.80 mg), *g* is the gravitational acceleration, $\rho_{\text{air}}$ is the air density (1.2 kg m^−3^), $v_{t}$ is the terminal velocity, and *A* is the projected area of all branches of the hairy barbs. The calculated values of *C*_D_ at different opening angles are summarized in table S1.

### Artificial intelligence

The authors used ChatGPT-5.3 to assist with English grammar editing. The authors take full responsibility for the content of the manuscript.

## Supplementary Materials

### The PDF file includes:

Figs. S1 to S28 Table S1 Notes S1 to S5 Legends for movies S1 to S11 References
