# Supplementary material for: Biohybrid chiral materials for an ultralight reconfigurable flying robot
Source: Sci Adv. 2026 Jul 23;12(30):eaef0912. doi: 10.1126/sciadv.aef0912 (PMC13394483; doi:10.1126/sciadv.aef0912)
Supplement: Supplementary file 1 — Figs. S1 to S28 Table S1 Notes S1 to S5 Legends for movies S1 to S11 References [file sciadv.aef0912_sm.pdf]

Science Advances
AAAS

Supplementary Materials for

Biohybrid chiral materials for an ultralight reconfigurable flying robot

Qi Yang *et al.*

Corresponding author: Dengfeng Li, dengfli2-c@my.cityu.edu.hk; Hao Zeng, hao.zeng@tuni.fi

Sci. Adv. **12**, eaef0912 (2026)
DOI: 10.1126/sciadv.aef0912

The PDF file includes:

Figs. S1 to S28
Table S1
Notes S1 to S5
Legends for movies S1 to S11
References

Other Supplementary Material for this manuscript includes the following:

Movies S1 to S11

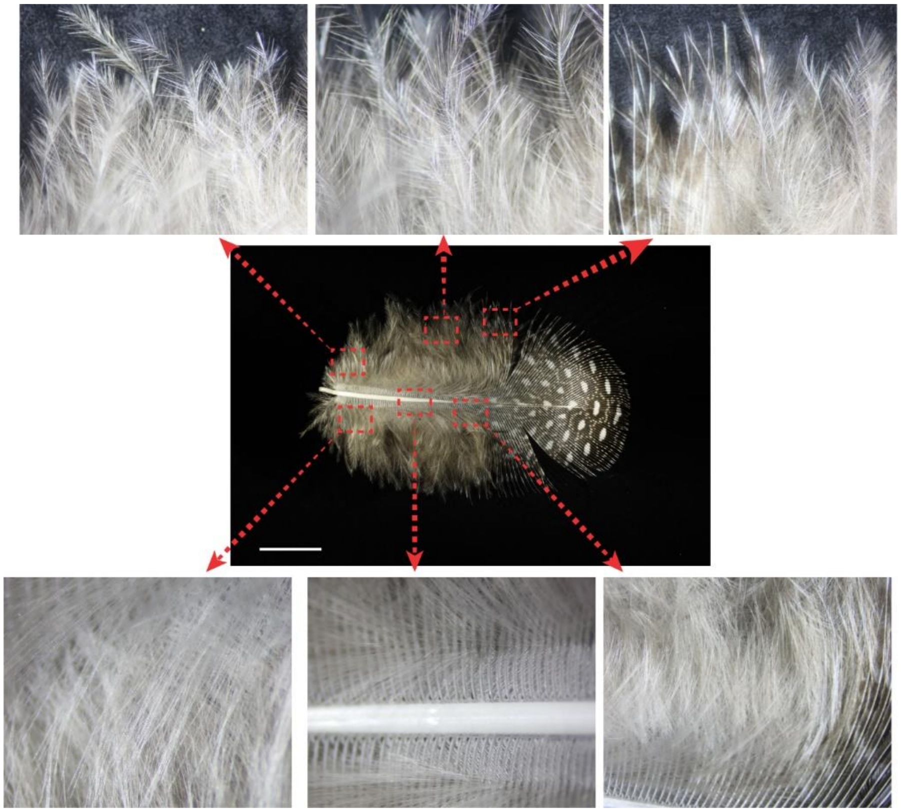

Fig. S1. Feather architecture. Photographs of a guinea fowl feather with zoomed-in images of different regions. Scale bar: 1 cm.

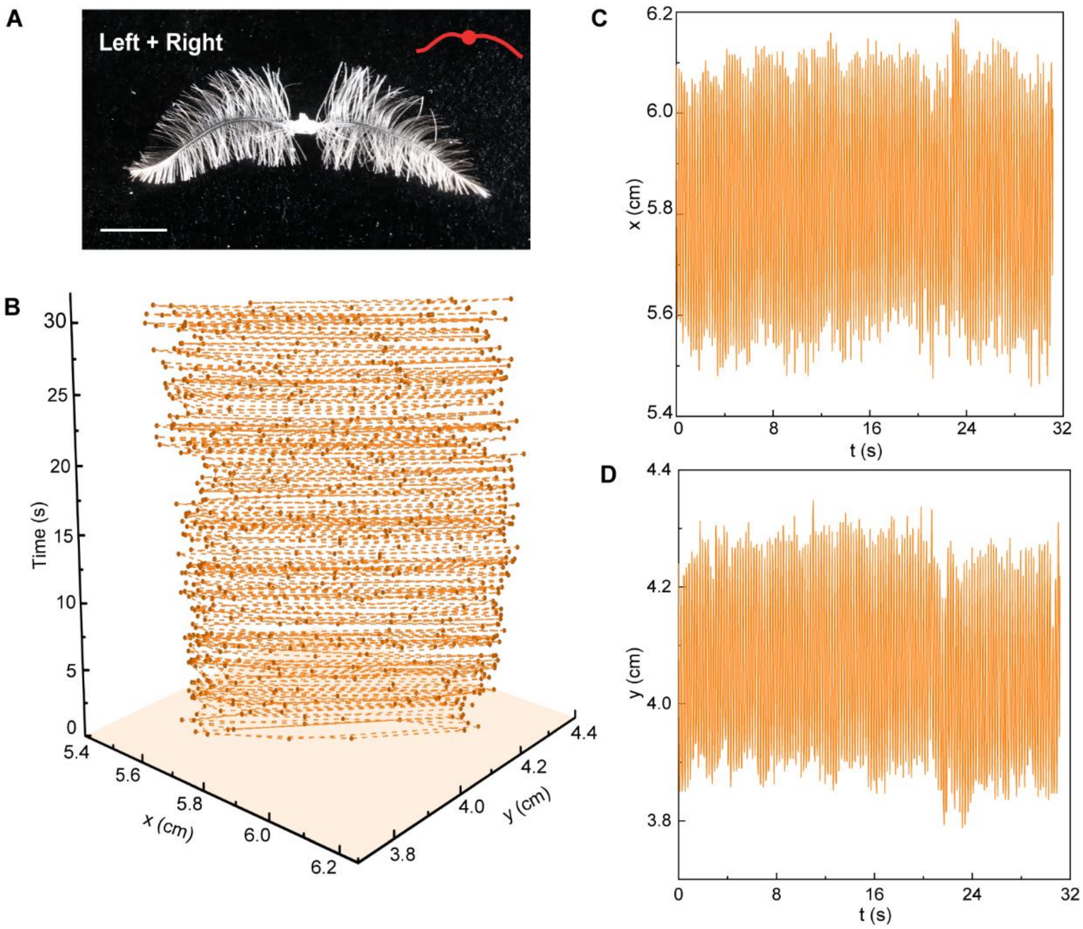

Fig. S2. Left-handed and right-handed assembly. (A) Photograph of a left-handed and right-handed afterfeathers assembly. (B) The gliding trajectory of the center of the assembly in the wind tunnel and the corresponding (C) X- and (D) Y-axial displacements as function of time. Scale bar: 5 mm.

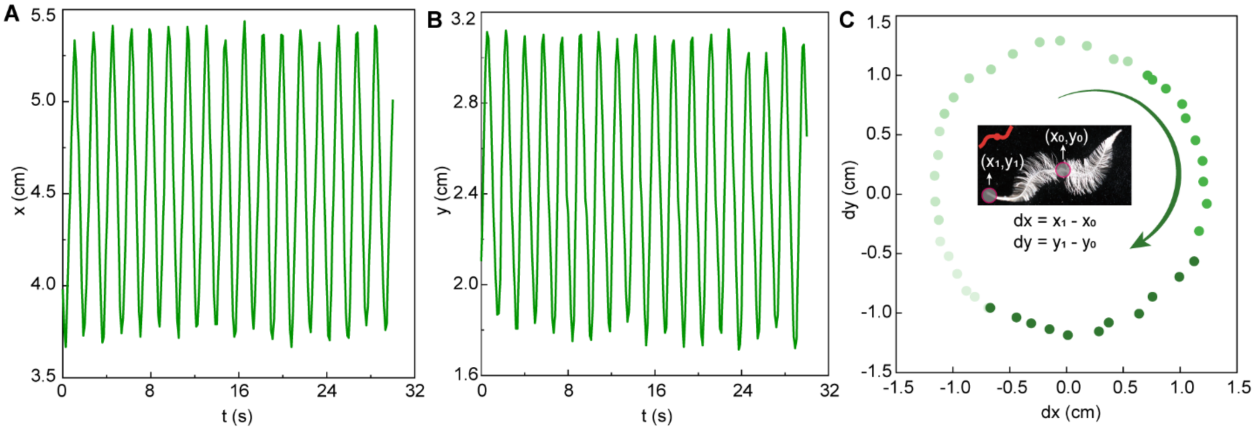

Fig. S3. Dynamics of right-handed and right-handed afterfeathers assembly. The (A) X- and (B) Y- coordinates of the geometrical center as a function of time. (C) The two-dimensional trajectory of one of the feather tips (x_1, y_1) with respect to the center position (x_0, y_0). The trajectory is plotted over one complete rotation.

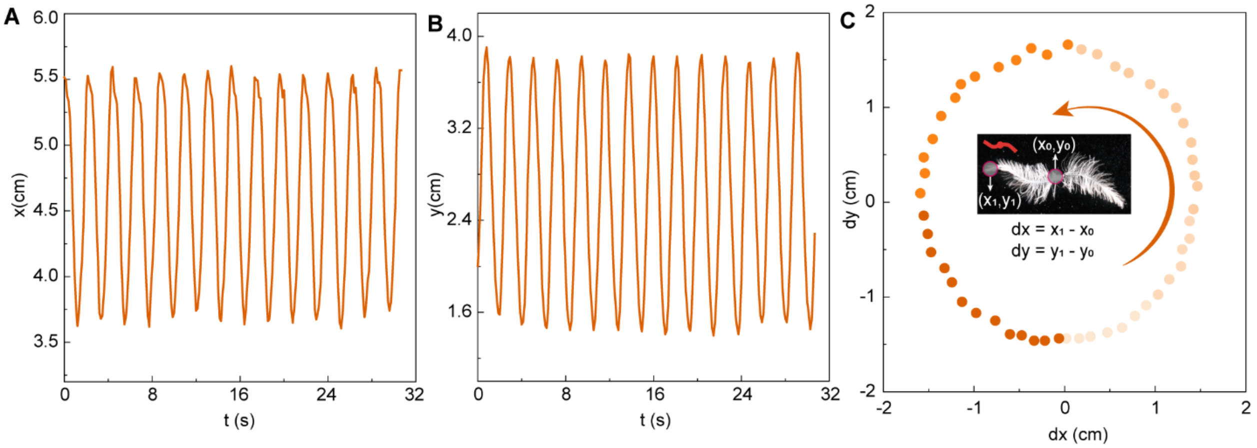

Fig. S4. Dynamics of left-handed and left-handed afterfeathers assembly. The (A) X- and (B) Y- coordinates of the geometrical center as a function of time. (C) The two-dimensional trajectory of one of the feather tips (x_1, y_1) with respect to the center position (x_0, y_0). The trajectory is plotted over one complete rotation.

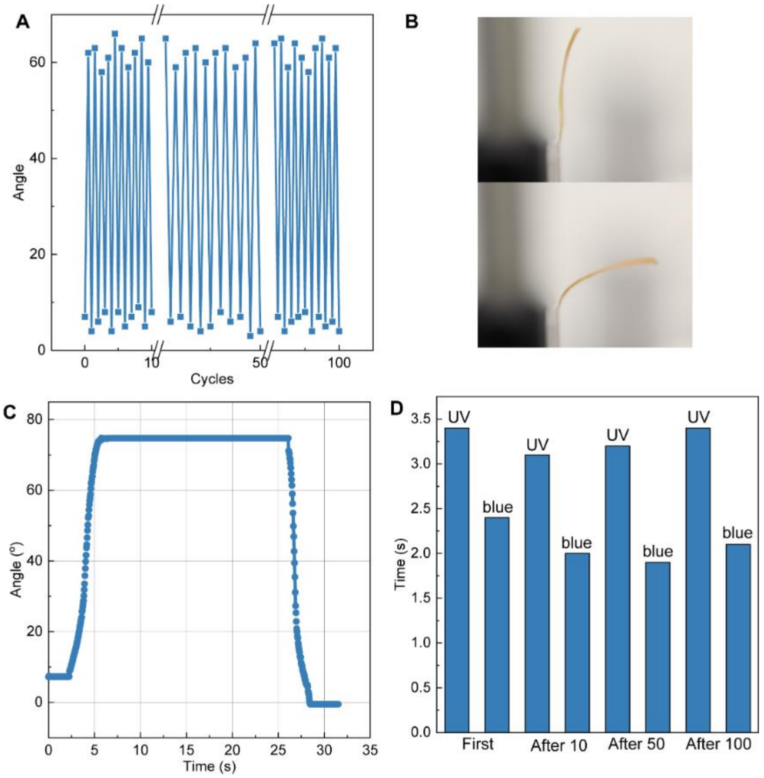

Fig. S5. Cycle test of the LCN actuator. (A) Bending angle of the LCN strip over one hundred light-actuation cycles. (B) Photographs showing the LCN strip before (top) and after UV excitation (bottom). (C) Deformation kinetics of the strip under UV and 460 nm illumination. (D) Response time of the LCN under UV and blue-light illumination after different cycle numbers. UV: 385 nm, 250 mW cm^{-2}; Visible light: 460 nm, 400 mW cm^{-2}. Strip size for material characterization: 2.1 cm × 0.3 cm × 20 mm.

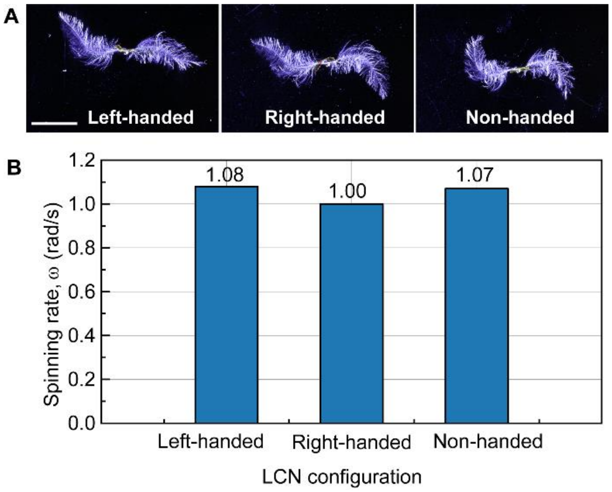

Fig. S6. The influence of central LCN on the aerodynamic behaviour. (A) Photographs of one identical feather robot by connecting two afterfeather with one thin filament. LCN strips with different configurations are mechanically stuck around the filament without introducing glue or other extra weight. Scale bar is 1 cm. (B) Spinning rate of the feather robot with different LCN configurations. The angular velocity (ω) was calculated from the average rotation period (T) obtained from video recordings using $\omega = 2\pi/T$. Each value represents the mean spinning rate computed from five consecutive rotations.

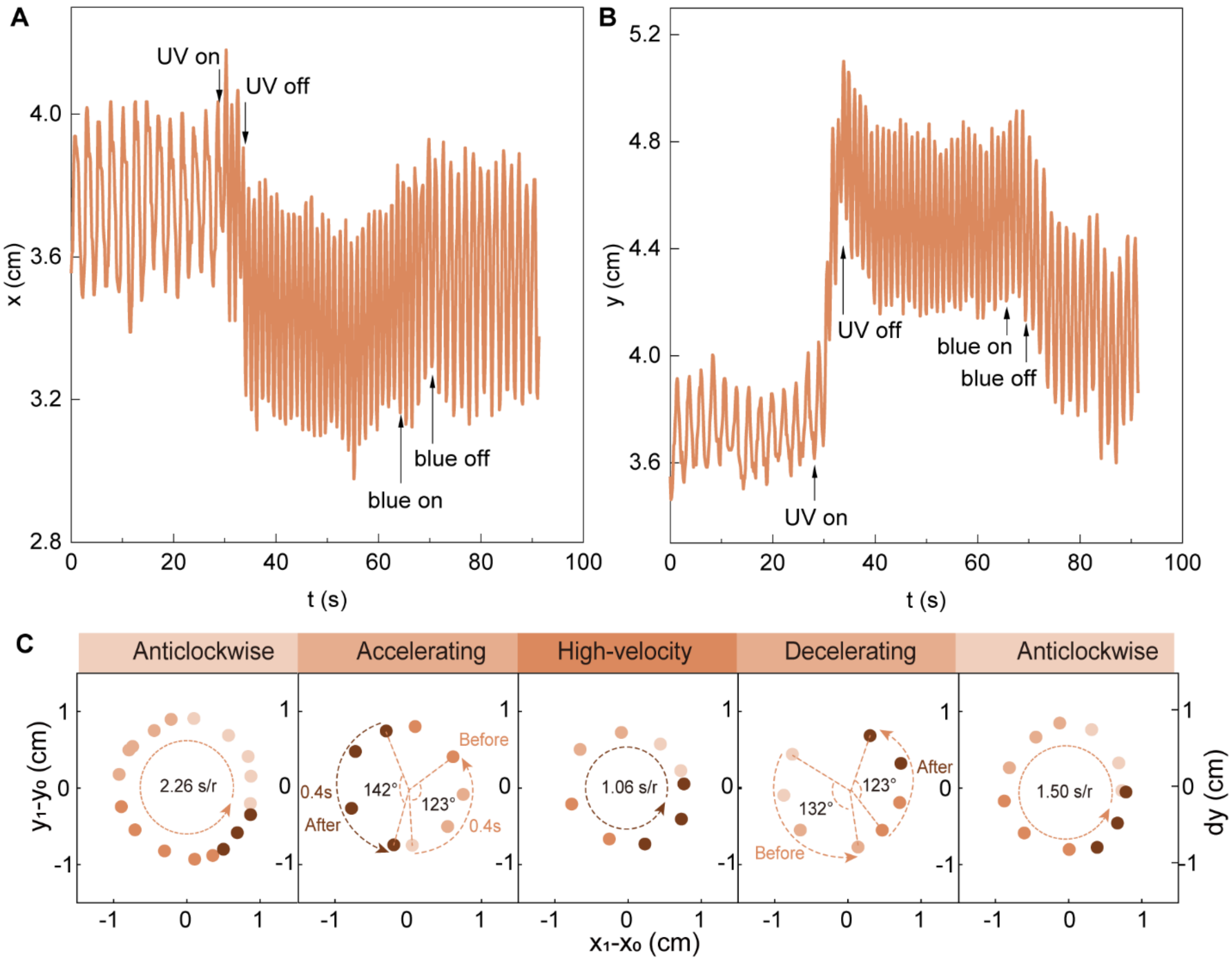

Fig. S7. Dynamics of left-handed afterfeathers and left-handed LCN assembly. The (A) X- and (B) Y- coordinates of the geometrical center as a function of time. UV: 385 nm, 150-200 mW/cm^2. Blue light: 460 nm, 100-170 mW/cm^2. (C) The two-dimensional trajectory of one of the feather tips (x_1, y_1) with respect to the center position (x_0, y_0) during different stages of light influence.

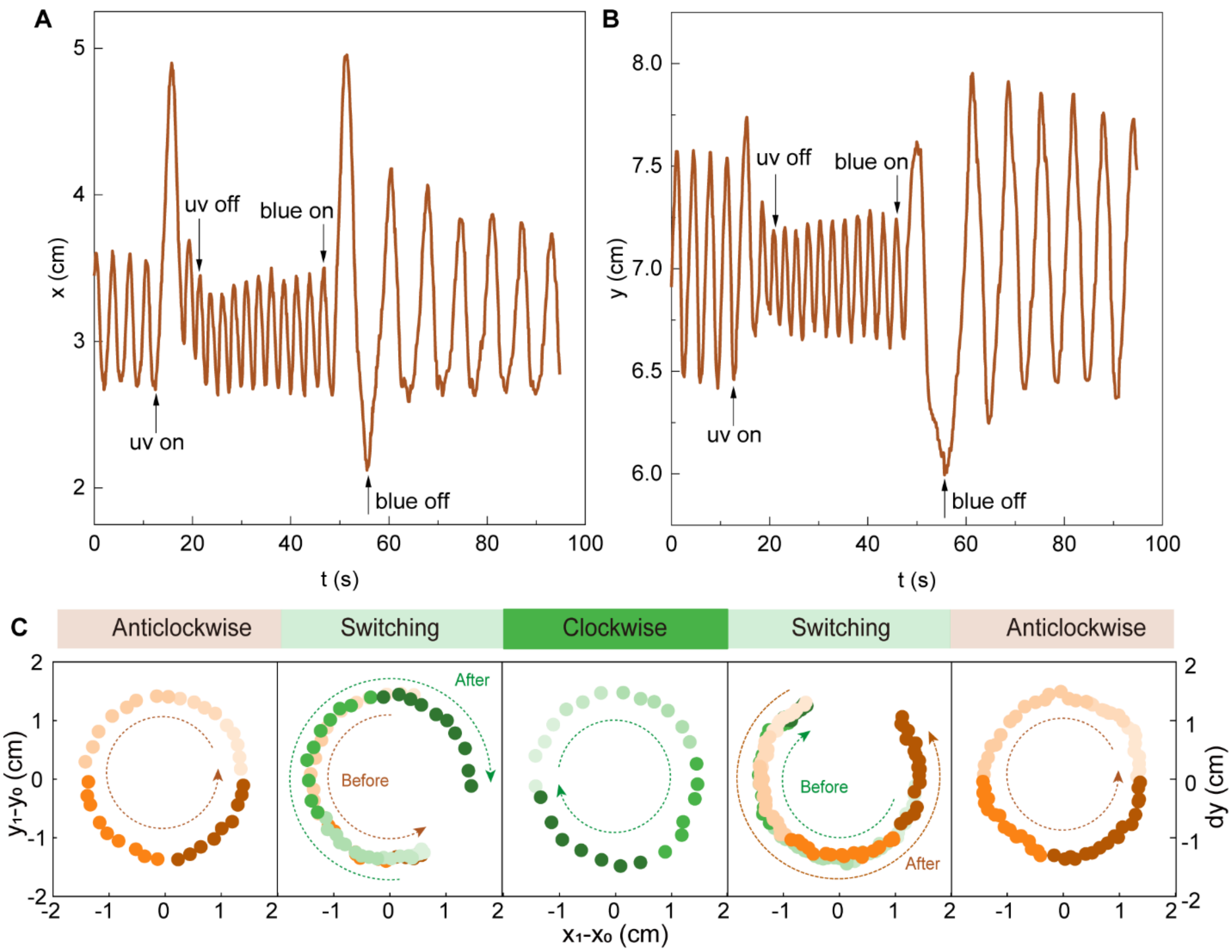

Fig. S8. Dynamics of left-handed afterfeathers and right-handed LCN assembly. The (A) X- and (B) Y- coordinates of the geometrical center as a function of time. UV: 385 nm, 150-200 mW/cm^2. Blue light: 460 nm, 100-170 mW/cm^2. C) The two-dimensional trajectory of one of the feather tips (x_1, y_1) with respect to the center position (x_0, y_0) during different stages of light influence.

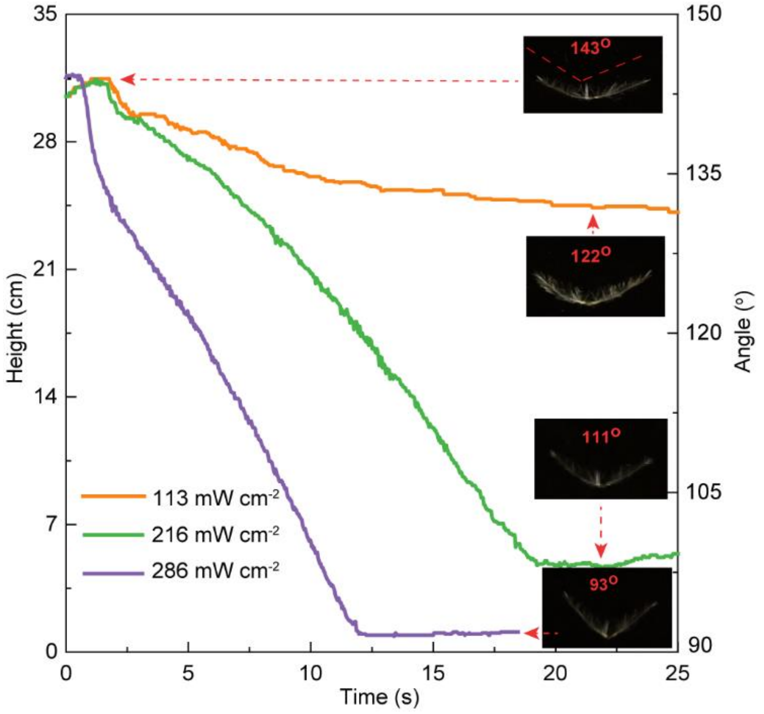

Fig. S9. UV light induced descend of flyers in the wind tunnel. With the increasing intensity of UV light, the opening angle of the actuator reduces resulting in falling inside the wind velocity gradient.

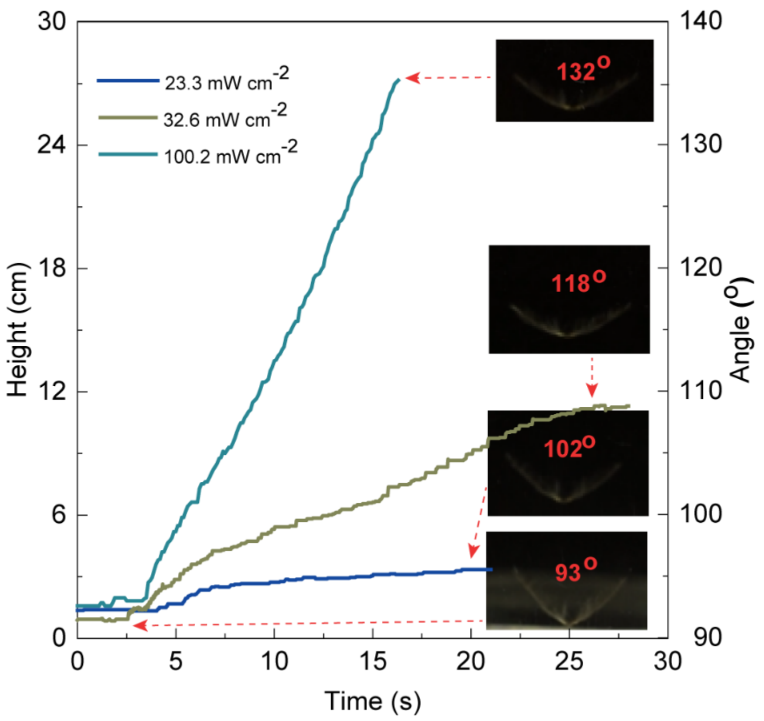

Fig. S10. Blue light induced elevation of the flyers in the wind tunnel. With the increase in blue light intensity the opening angle of the actuator increases leading to the elevation inside the wind velocity gradient.

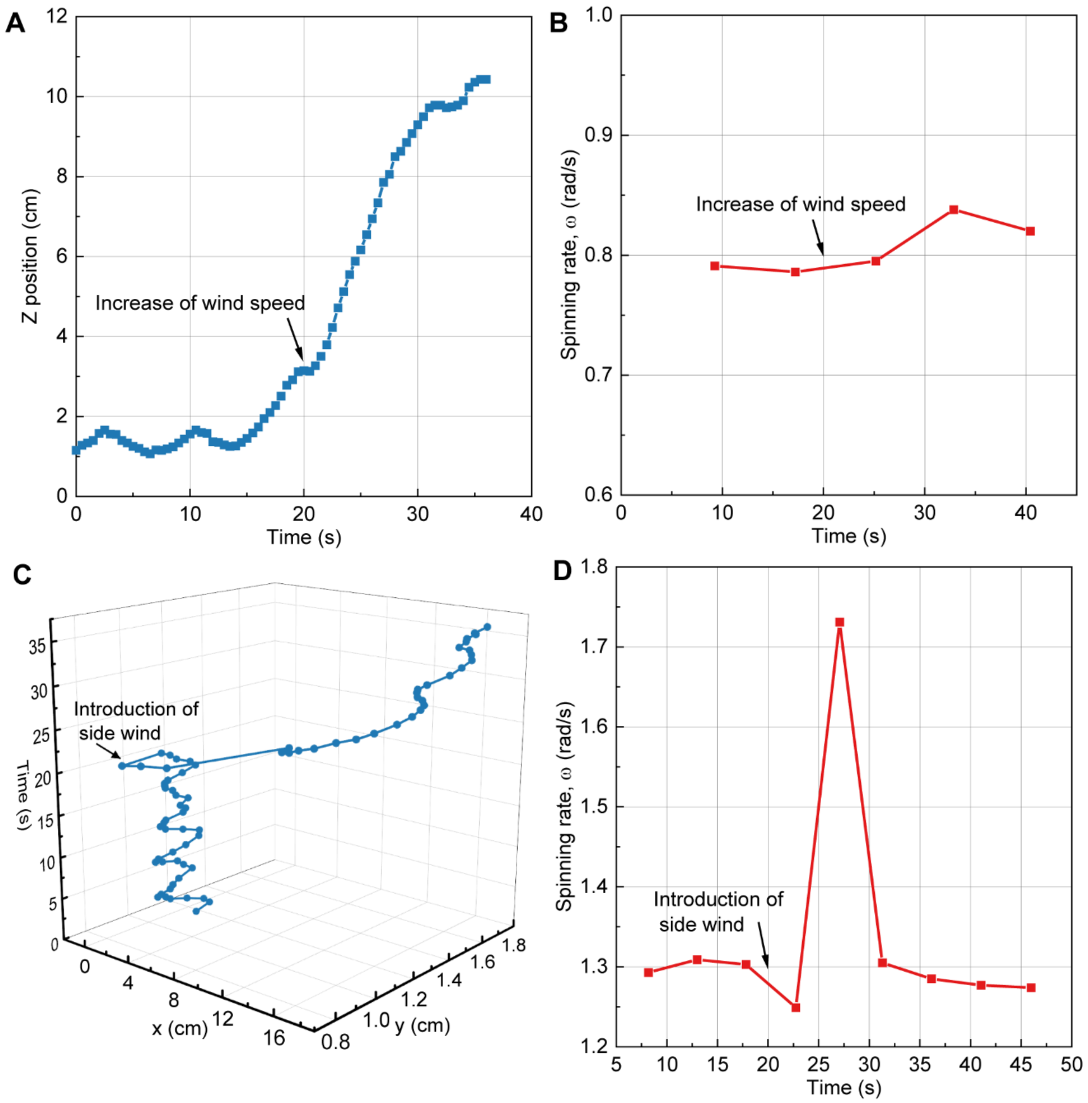

Fig. S11. The effect of wind disturbance on flyer performance. (A) Change in flyer height following a sudden increase in wind speed. (B) Variation in spinning rate after the sudden increase in vertical wind speed. The initial wind speed is 0.22 m/s, which increases to 0.27 m/s at 20 s and remains at that level until the end. (C) Lateral position change following the introduction of a side wind. (D) Variation in spinning rate under the influence of a side wind. The vertical wind speed remains constant at 0.26 m/s. A side wind of 1.18 m/s is introduced at 20 s and continues until the end.

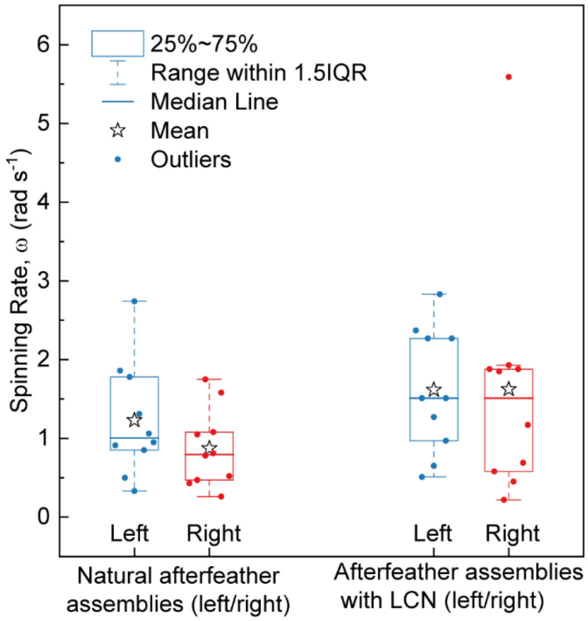

Fig. S12. Distribution of spinning rate. The experiments were performed in two groups. Group 1: natural afterfeather individual pieces, including 10 left-handed and 10 right-handed samples. Group 2: natural afterfeather–LCN hybrid flyers, in which the afterfeathers include 10 left-handed and 10 right-handed samples. Data are shown as mean ± s.d. (n = 10 independent samples).

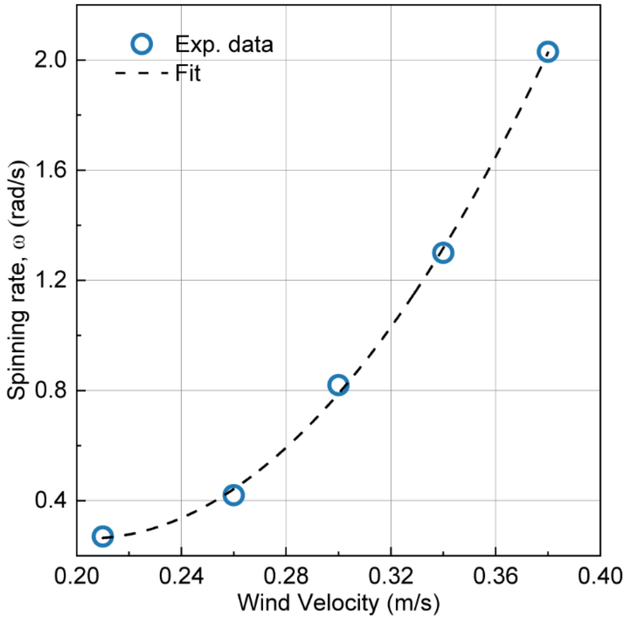

Fig. S13. Relationship between spinning rate and wind velocity. The vertical wind tunnel is operated at different flow velocities, which are measured at a fixed height (3 cm above the honeycomb) using an anemometer. The flyer is photoreconfigured to maintain mid-air rotation at the same height as the wind speed measurement. The spinning rate (ω, in rad/s) is calculated as $\omega = 2\pi/T$, where T denotes the time required to complete one full rotation cycle.

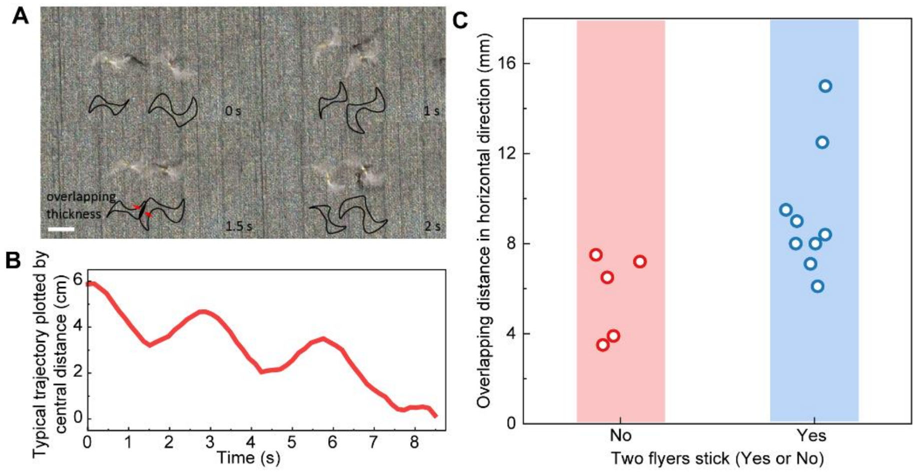

Fig. S14. Critical distance for mid-air interaction. (A) Top-view snapshots of two flyers spinning at close proximity. The overlapping distance (the thickness) is defined as the maximum horizontal overlap between the two feathers. Scale bar is 2 cm. (B) Representative trajectories based on the center-to-center distance between the two flyers. (C) Occurrence of auto-connection events as a function of overlapping distance.

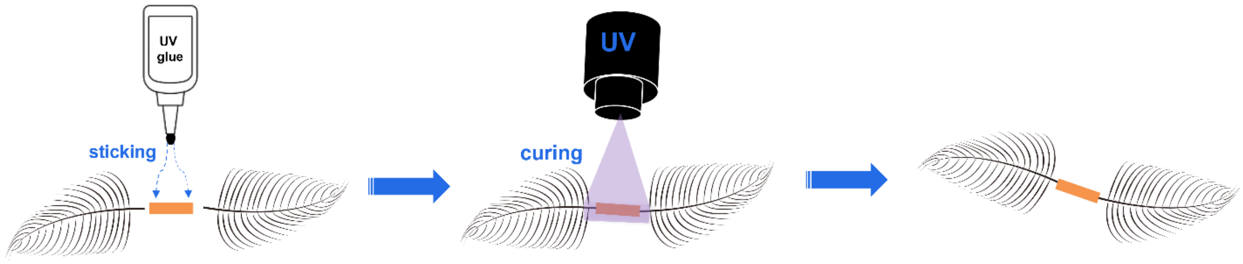

Fig. S15. Illustration of the preparation processes of feather-actuator assembly. Two afterfeathers are attached to liquid crystal actuator strip using UV curable glue.

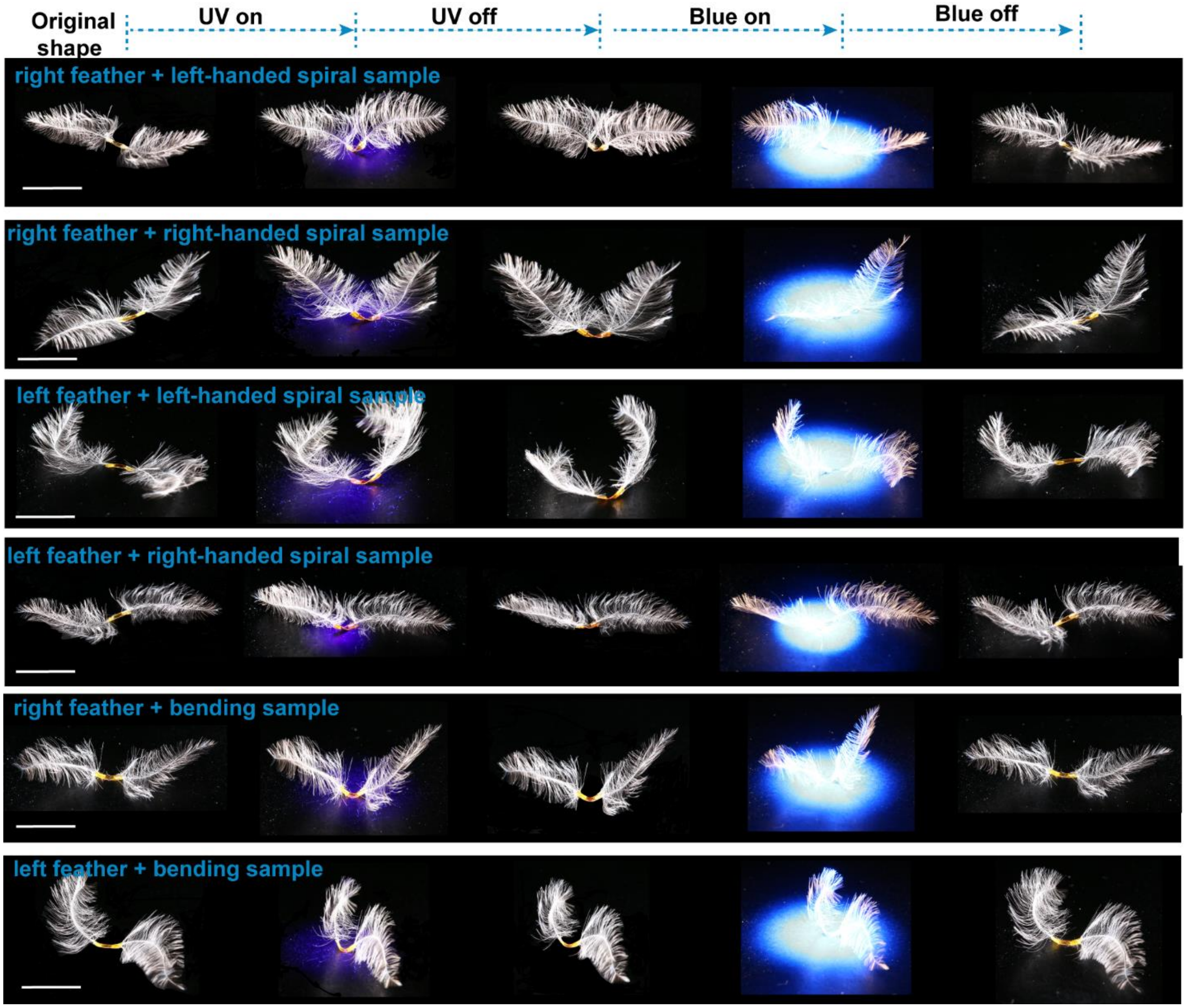

Fig. S16. Photomechanical deformation in different feather-actuator assemblies. Six types of assemblies, i.e., right-handed afterfeathers-left-handed LCN, right-handed afterfeathers-right-handed LCN, left-handed afterfeathers-left-handed LCN, left-handed afterfeathers-right-handed LCN, and right-handed afterfeathers-bending LCN and left-handed afterfeathers-bending LCN, upon illumination of UV and Blue light exposure. Scale bars are 1 cm. UV: 385 nm, 200 mW/cm^2; Blue: 460 nm, 157 mW/cm^2.

Opening angles (°)	Terminal velocity (m/s)	Projected area (mm^2)	Drag coefficient
178	0.23	185.0	1.30
126	0.26	164.8	1.16
95	0.28	136.4	1.19
90	0.31	130.8	0.99
65	0.38	99.40	0.87

Table S1. Drag coefficient at different opening angles.

Supplementary Note 1: Chirality and porosity analysis of natural afterfeathers

1.1 Variability of afterfeather samples

Naturally afterfeathers emerging from either side of the central shaft (rachis) display two clearly distinct morphological forms, characterized as left-handed and right-handed. Considerable variability is observed even among afterfeathers originating from the same side of the shaft, with pronounced differences in shape, size, and the spatial distribution and density of their constituent filaments. Photographs illustrating this variability among ten left-handed and ten right-handed afterfeathers are shown in Fig. S17 and Fig. S18 respectively. Because of such variability feather robots made of the same handedness afterfeathers show quantitative differences in rotational motion in this manuscript.

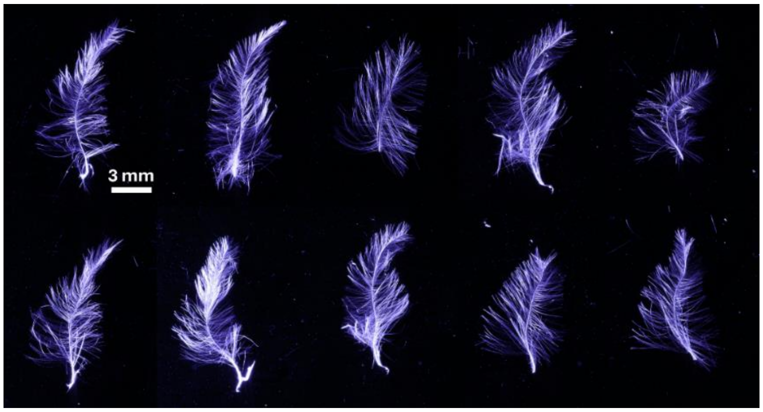

Fig. S17. Left-handed afterfeathers. Photographs of ten left-handed afterfeathers collected from different regions of a single feather.

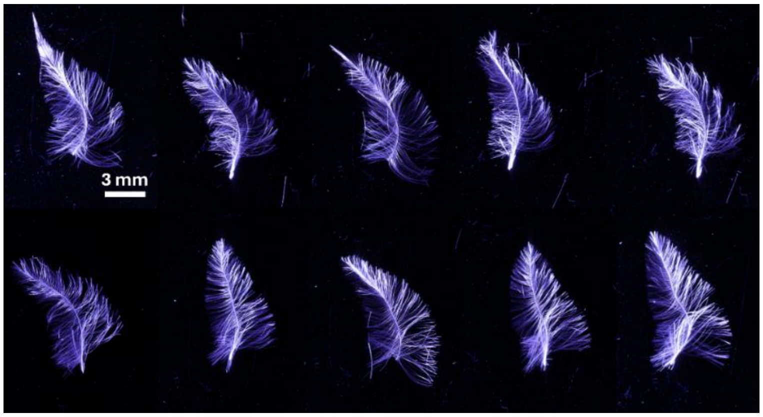

Fig. S18. Right-handed afterfeathers. Photographs of ten right-handed afterfeathers collected from different regions of a single feather.

1.2 Quantification of the chirality of the afterfeathers

Left- and right-handed afterfeathers exhibit opposite rotational motions when subjected to airflow in a wind tunnel, indicating the presence of an inherent structural bias. This bias can be understood in terms of chirality, which reflects a preferred handedness in form and function. Closer examination reveals that each afterfeather comprises two primary components: the central vane that is indicated by the red dashed line in Fig. S19A, and arrays of filaments extending from either side of this vane. The spatial arrangement and asymmetry of these filaments relative to the central vane likely contribute to the observed chiral behavior.

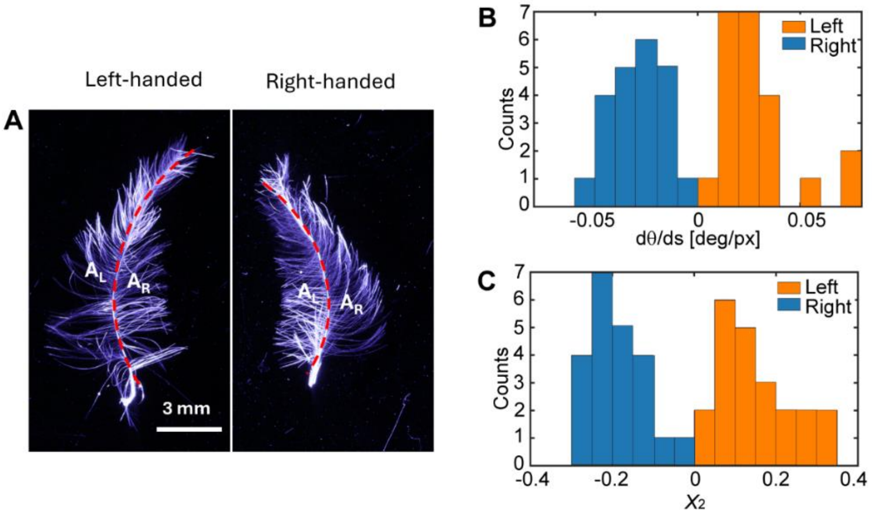

Fig. S19. Chirality of afterfeathers. (A) Representative left- and right-handed afterfeathers, with dashed lines indicating the central vane. Chirality is quantified using two independent measures: (B) the mean angular dispersion of barbs relative to the vane, capturing curvature-induced asymmetry, and (C) the normalized area difference between the two sides of the vane, reflecting lateral structural bias. Distributions are shown for 22 afterfeather samples, highlighting substantial variability while preserving consistent handedness distinctions.

The central vanes are not straight but inherit a pronounced curvature. Notably, the left- and right-handed afterfeathers exhibit opposite dominant bending directions, which can be interpreted as one form of manifestation of chirality. We quantify the two-dimensional projection of the curvature from the images of afterfeathers by evaluating the average angular dispersion $\left(\frac{d\theta}{ds}\right)$ along the vane of the afterfeathers. Thus, we can define an average chirality parameter as, $\chi_1 = \frac{d\theta}{ds}$. For the representative left- and right-handed afterfeather image shown in Fig. S19A, we obtain: $\chi_1 = (0.0262 \pm 0.0005)$ deg/px and $\chi_1 = (-0.0276 \pm 0.0014)$ deg/px. The opposite sign re-emphasizes the contrasting bending directions.

In addition to vane curvature, a secondary source of asymmetry arises from the distribution of filaments area on either side of the vane. This can be quantified as: $\chi_2 = \frac{A_L - A_R}{A_L + A_R}$, where A_L and A_R denote the areas on the left and right sides of the vane, respectively. By this definition, $\chi_2 > 0$ indicates left-side dominance, whereas $\chi_2 < 0$ indicates right-side dominance. For the same afterfeathers shown in Fig. S19A, we obtain $\chi_2 \simeq +0.199$ and $\chi_2 \simeq -0.034$.

Taken together, the combined effects of vane curvature (χ_1) and lateral area asymmetry (χ_2) establish an inherent chirality in afterfeathers. To assess the statistical nature of this chirality, we measured $\chi_1$and χ_2 across 22 different afterfeather samples. The distributions are shown in Fig. S19B and S19C, respectively. While the magnitudes of both parameters vary widely, indicating substantial sample-to-sample variability, the qualitative distinction between left- and right-handed forms remains robust.

1.3 Porosity analysis of afterfeathers

The porosity of the afterfeathers plays a critical role in governing their aerodynamic behaviour under airflow. In particular, the distribution of void spaces within the feather structure influences drags, permeability, and the interaction between air and the filament network. To characterize this structural property, we quantify the projected porosity of the afterfeathers based on image analysis.

Closer examination reveals that each afterfeather consists of a network of filamentary structures forming a porous architecture with distributed air gaps. These void regions are interspersed between the solid fibers and collectively determine the effective porosity of the structure. The spatial distribution and density of these voids are expected to influence airflow penetration and, consequently, the dynamic response of the afterfeathers.

To quantify this effect, we evaluate the two-dimensional projected porosity from optical images of the afterfeathers. The porosity is defined as the ratio of void area (space between the filaments) to the total projected area of the structure, $\phi = \frac{A_{\text{void}}}{A_{\text{total}}}$, where A_{void} represents the area occupied by void regions and A_{total} denotes the total region of interest enclosing the afterfeather.

The porosity was computed using an image-processing workflow implemented in ImageJ. The acquired images (as exemplified in Fig. S20A) were first converted to 8-bit grayscale format to enable intensity-based segmentation (Fig. S20B). A region of interest (ROI) corresponding to the afterfeather was then selected, and the surrounding background was removed to avoid bias. Thresholding was applied to identify the solid filament regions. The solid area fraction within the ROI was quantified, and the porosity was obtained as the complement of the solid fraction, $\phi = 1 - \frac{A_{\text{solid}}}{A_{\text{total}}}$, expressed as a percentage.

To evaluate sample-to-sample variability, the projected porosity was measured for multiple afterfeathers. The porosity values for five left-handed and right-handed afterfeather structures are summarized in Fig. S20C.

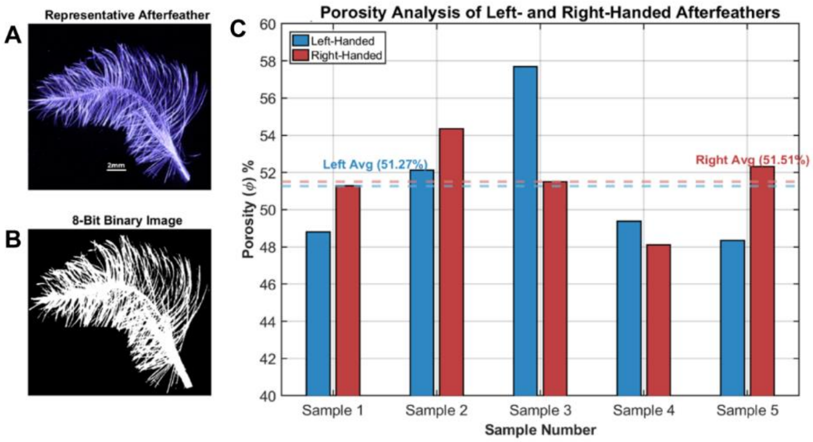

Fig. S20. Porosity of afterfeathers. The porosity value of an afterfeather sample is quantified by processing its optical image to 8-bit binary image. (A) The original optical image of a representative afterfeather. (B) The associated 8-bit binary image obtained through image thresholding distinguishably separates the void region from the solid filaments. (C) The porosity values of five left- and five right-handed afterfeathers. Each bar represents the measured porosity of an individual sample, while dashed and dash-dotted horizontal lines indicate the average porosity of the left- and right-handed afterfeathers.

As illustrated in Fig. S20C, both left- and right-handed afterfeathers exhibit comparable porosity distributions, with average values around 51%. Although individual samples show noticeable variation, likely due to intrinsic structural heterogeneity and sensitivity to image thresholding, no significant difference in porosity is observed between the two chiral groups.

Supplementary Note 2: Wind tunnel setup

To investigate the gliding behaviors of the assemblies, two custom-built wind tunnel systems were used, namely a small-scale wind tunnel and a large-scale wind tunnel as shown in Fig. S21. Both systems share the same fundamental design, with the primary difference being their dimensions and construction materials. The airflow was generated by fans installed at the base of the tunnel, producing an upward flow. To ensure a stable and uniform flow field, multiple layers of flow straighteners, including honeycomb structures and fine meshes, were positioned above the fan to eliminate vortices and smooth the airflow before entering the test section. The walls of the test section were fabricated from transparent PET films in the small tunnel and acrylic plates in the large tunnel, which not only ensured stable flow conditions but also provided high optical transparency for visualization. The airflow velocity was regulated by an Arduino Uno control system, enabling adjustment of the upward flow during experiments.

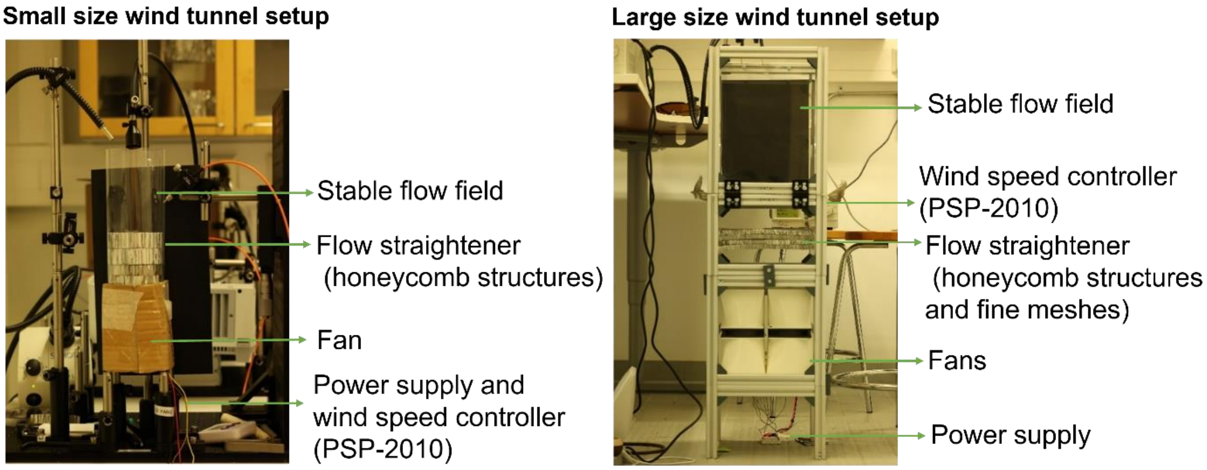

Fig. S21. Photographs of wind tunnel construction. The wind tunnels are mainly composed of fans, flow straighteners, and frames.

To characterize the vertical distribution of airflow in the large-scale wind tunnel, the wind speed was measured along the height of the test section. Fig. S22 shows a gradual decrease of wind speed from the bottom to the upper outlet of the tunnel.

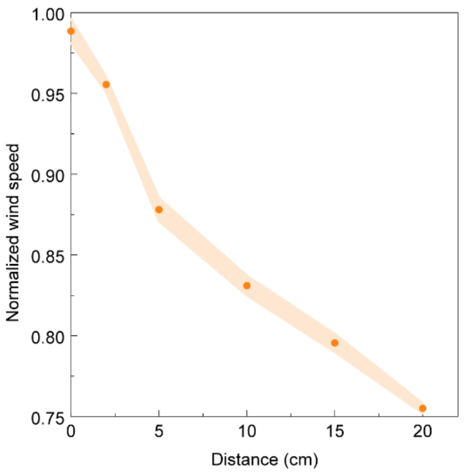

Fig. S22. Wind velocity gradient measurement. The speed is measured at different height inside the test section, as a function of distance to the inlet. Inlet wind speed (distance = 0) is 0.26 m/s. Data are shown as mean ± s.d. (n = 3 independent samples).

Supplementary Note 3: Preparation of liquid crystal network (LCN)

The actuating films used in this work were based on LCN (*24*). The fabrication began with the construction of a hybrid-aligned liquid crystal cell. Two glass substrates were coated with different alignment layers: one substrate was spin-coated with 5 wt% PVA aqueous solution, baked at 90 °C for 20 min, and then unidirectionally rubbed with a satin cloth to induce planar alignment. The other substrate was coated with a vertical-aligning polyimide and baked at 180 °C for 20 min. The substrates were assembled into a sandwich cell using UV-curable adhesive, with 20 μm borosilicate glass microspheres as spacers to define the cell thickness. The reactive mesogen mixture consisted of ST03866 (52 mol%), ST02670 (18 mol%), ST00975 (21 mol%), ST04181 (6 mol%), and photoinitiator 819 (1.5 mol%). The mixture was dissolved in dichloromethane, and after complete solvent evaporation at 90 °C, the molten mixture was filled into the cell via capillary action. The filled cell was cooled at a rate of 3 °C/min to 50 °C and then photopolymerized under 420 nm light (20 mW cm^{-2}) for 30 min. Finally, the cell was carefully opened to obtain a free-standing LCN film for further experiments. Details of the molecular structures and preparation steps are shown in Fig. S23.

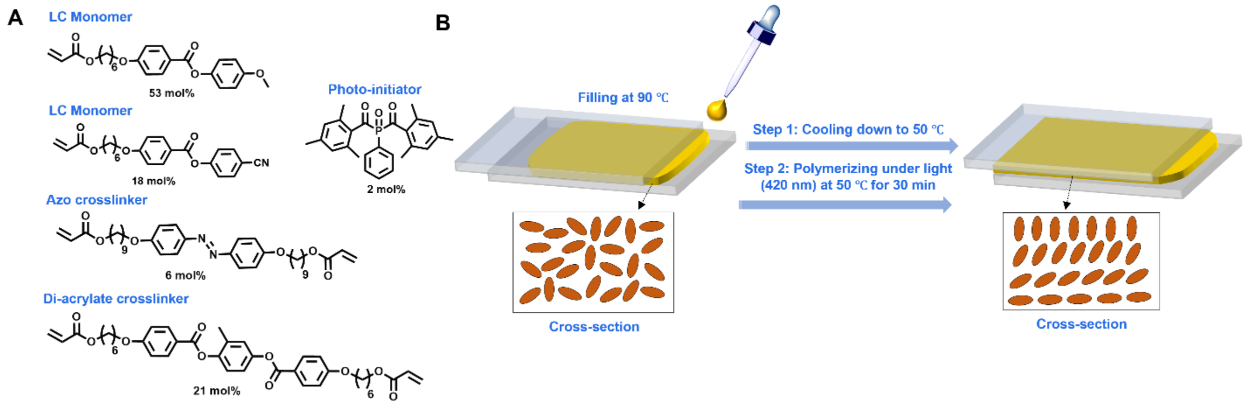

Fig. S23. The preparation processes of LCN. (A) The chemical compounds used in this study. (B) Preparation steps of LCN and the illustration of associated alignment of liquid crystal molecules.

By cutting the films along different orientations with respect to the liquid crystal director, three distinct actuators with different deformation modes were obtained. They are (1) bending actuator by cutting along the director, (2) left-handed torsional actuator by cutting at +45° with respect to the director and (3) right-handed torsional actuator by cutting at -45° with respect to the director. The cutting directions are schematically shown in Fig. S24.

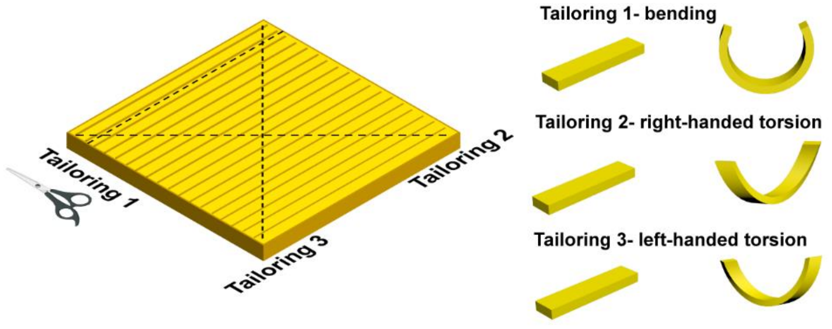

Fig. S24. Different deformation modes in LCN actuators. Depending on the cutting angles, bending, left- and right-handed torsion are obtained from the LCN actuator strips.

Supplementary note 4: Computational Fluid Dynamics (CFD) simulation.

4.1. Simulation method.

Numerical simulations were performed using ANSYS Fluent 2023 R2. The Reynolds number based on the characteristic feather length in the wind tunnel was estimated to be approximately 140, while the Reynolds number based on the filament diameter was on the order of unity. These estimates indicate that the flow remains predominantly in the laminar regime at both the macroscopic feather scale and the microscopic filament scale. Accordingly, a laminar flow model was employed to simulate the airflow around the feather structure.

The simulated feather pair consists of two identical feathers. Each feather comprises a stiff central shaft with 92 smaller barbs attached along its length. The shaft has a diameter of 0.1 mm, while each barb has a diameter of 0.03 mm. The two feathers are connected at their bases by a flat LCN film with dimensions of $1.2 \times 0.3 \times 0.02$ mm³. The overall dimensions of the assembled feather pair are $22.04 \times 9.98 \times 5$ mm³. The geometric design of the feather–LCN assembly from multiple viewing angles is shown in Fig. S25.

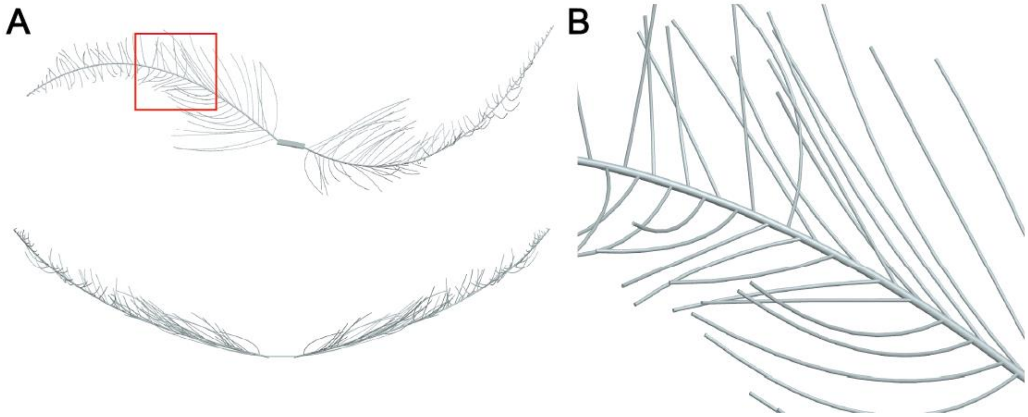

Fig. S25. The architecture of feather-LCE assembly. (A) The entire assembly structure taken from top-view (top) and side-view (bottom) angles. (B) Zoomed-in design images of the shaft and barbs (referred to the red box area in A).

To obtain the pressure distribution on a representative section, a slicing plane was defined within the computational domain at a height of 1.75 mm above the base of the feather pair. The pressure field from the CFD simulations was then mapped onto this plane using the post-processing tools in ANSYS Fluent. Pressure values were interpolated from the surrounding mesh cells onto the slicing plane to generate pressure-contour plots, which visualize the sectional pressure distribution shown in Figs. 2F, 3C, and 3J of the main text.

The flower model (Figs. 5D–G) was approximated in the CFD simulations as a hemispherical surface with a diameter of 40 mm, while the larger feather (Figs. 5H–J) was represented as a curved, impermeable plate with a thickness of 1 mm. Pressure-contour plots (Fig. 5I) were obtained following the same procedure described above. Flow pathlines were

generated by seeding massless tracer particles into the airflow and tracking their trajectories over time using the post-processing tools in ANSYS Fluent (Fig. 5F).

4.2 Aerodynamics torques and convergence criteria.

Figure S26 shows the aerodynamic torque acting on an afterfeather–LCN assembly fixed inside the wind tunnel. At time = 0 s, a uniform airflow with a velocity of 0.15 m/s is imposed at the inlet boundary. The torque for right-handed afterfeather–LCN assembly converges to 9.81×10^{-11} N·m, whereas the torque acting on the left-handed assembly is -9.65×10^{-11} N·m. The torque magnitudes are nearly identical but of opposite sign, indicating that the chirality of the structure dictates the direction of the generated aerodynamic torque. The slight discrepancy in magnitude is attributed to limitations in mesh resolution when resolving the extreme geometric multiscale nature of the system.

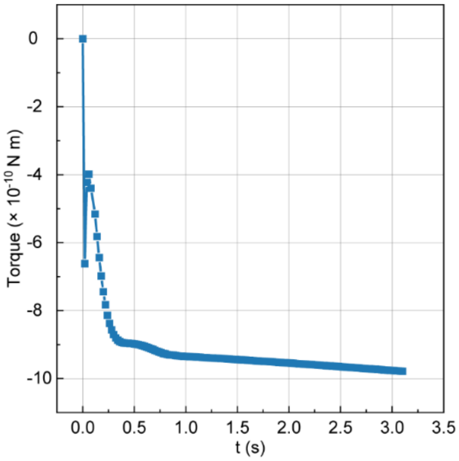

Fig. S26. Time evolution of the aerodynamic torque acting on the left-handed feather pair from numerical simulation.

Movie S11 shows the temporal evolution of the pressure field following the onset of airflow. In all simulations, convergence was assessed by monitoring the continuity residual. Convergence was achieved when the difference between successive iterations fell below 1×10^{-6} in the value of continuity residual, indicating that the solution had reached a steady, converged state.

4.3 Transient rotational dynamics of the feather pair.

To investigate the rotational dynamics of the handed afterfeather-LCN assembly, a transient simulation was performed. For comparison, a free-falling experiment was performed by releasing the assembly within a stead wind, and the temporal evolution of angular velocity was recorded.

All simulation settings were kept consistent with those used in the steady-state analysis. The density of the feather structure was set to 40 kg m^{-3}. A dynamic mesh combined with a six-degree-of-freedom (6DOF) solver was enabled to allow rigid-body motion of the structure.

Only the rotational degree of freedom about the gravity direction (R_z) was released, while all remaining translational and rotational degrees of freedom were constrained.

The transient simulations reveal the temporal evolution of the aerodynamic torque and the resulting angular velocity of the feather assembly (Fig. S27B–C). As shown in the figure, the aerodynamic torque increases rapidly at the onset of motion and reaches a maximum value of 3.5×10^{-10} N·m at approximately 0.02 s. The torque then decreases sharply, with the rate of decrease gradually slowing, and approaches a nearly constant value of approximately 2×10^{-12}N·m after about 0.05 s. Correspondingly, the angular velocity increases continuously over time, albeit with a progressively reduced growth rate, reaching approximately 1.6 rad s^{-1} at 0.3 s. These results are consistent with the expected rotational dynamics, in which the angular velocity is governed by the applied torque and the rotational inertia of the system.

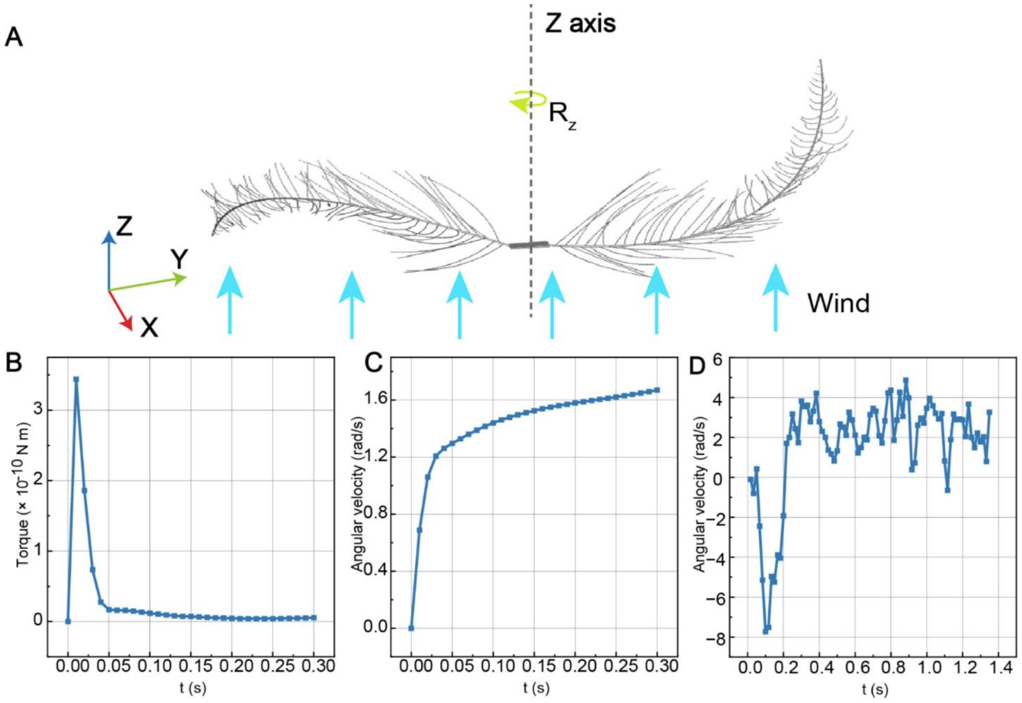

Fig. S27. Torque and angular velocity of right-handed feather pair. (A) Simulated torque acting on the right-handed feather pair. (B) Simulated torque acting on the right-handed feather pair. (C) Simulated angular velocity of the right-handed feather pair. (D) Angular velocity measured in the free-fall experiment.

Experimentally, the rotational velocity of a freely falling feather was also measured (Fig. S27D). During the first 0.2 s, the angular velocity exhibits rapid fluctuations, primarily due to disturbances introduced by manual release of the specimen. After this initial perturbation phase, the angular velocity increases to approximately 3 rad s^{-1} within the subsequent 0.1 s and then oscillates around this value. Notably, the rotational speed differs between the simulations and experiments, which is attributed to the substantial natural variability among feather samples. Nevertheless, the characteristic transient timescale is on the order of 0.1 s in both the simulated and experimental cases, indicating qualitative consistency between the two.

Supplementary Note 5: Torque reversal due to structural reconfiguration.

Light-induced twisting of the LCN connector linking two afterfeather assemblies leads to a reversal in the rotational direction in the midair motion. To gain insight into the underlying fluid-structure interactions governing such behavior, we performed CFD simulations, modeling the afterfeathers as a simplified planar structure with prosity. The simulations were carried out using the laminar flow module in ANSYS Fluent, with inlet wind flow velocity as $v_{inlet} = 0.15$ ms^{-1}. The schematic representations of the modeled geometry, shown in both top and side views, are presented in Fig. S28A.

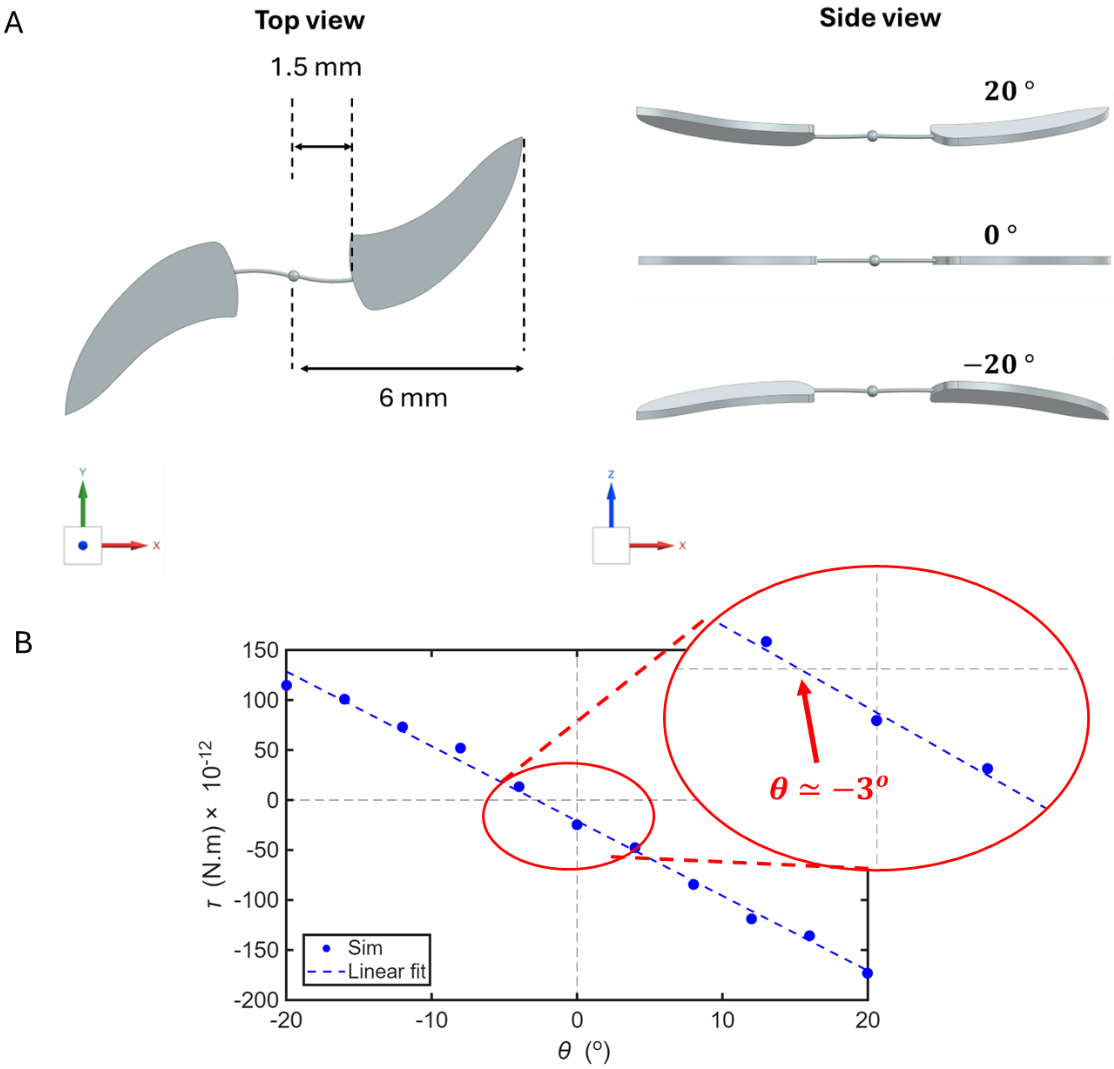

Fig. S28. Aerodynamic simulation of torque reversal. (A) The top view and side view of the approximate planer model of afterfeather robot at three different twist angles 0°, 20° and -20°. (B) The net torque acting on the robotic structure as a function of twist angle exhibits a positive to negative transition across -3° twist angle.

For such porous structures, the pressure drop across the plate can be described by the Darcy–Forchheimer equation: $\frac{\Delta P}{L} = \frac{\mu}{\alpha} v + C_2 \frac{1}{2} \rho v^2$, where ΔP is the pressure-drop across porous structure of thickness L, v is the fluid velocity, μ is the dynamic viscosity, ρ is the fluid density (*44, 45*). The coefficients $\frac{1}{\alpha}$ and C_2 are the viscous and inertial resistance coefficients, respectively. The first term in the above equation captures the viscous contribution and the second term accounts for the inertial contribution. In our study case, in laminar flow regime the viscous contribution is the dominant factor.

Direct determination of these resistance coefficients for the complex afterfeather geometry is non-trivial. Instead, we approximate them using a porous-bed model, in which the resistances are expressed $\frac{1}{\alpha} = \frac{150(1-\epsilon)^2}{D_p^2 \epsilon^3}$ and $C_2 = \frac{3.5(1-\epsilon)}{D_p \epsilon^3}$, where ϵ is the porosity of the structure and D_p is the pore-size. From two-dimensional image analysis shown in Supplementary Note 1, we estimate an average porosity of $\epsilon \simeq 0.51$. The pore size is approximated as, $D_p = \frac{l}{n}$, where $n = 18$ is the number of filaments within length scale $l = 3$ mm. Based on above, the viscous and inertial resistances are obtained as, 8.85×10^9 m^{-2} and 7.17×10^3 m^{-1} respectively.

Using these parameters, we performed aerodynamic simulations. Owing to the asymmetric geometry of the structure, the resulting aerodynamic field exerts a non-zero tangential force, which generates an aerodynamic torque and drives the structure into a rotational state. The net aerodynamic torque acting on the structure as a function of twist angle is shown in Fig. S28B. At zero twist angle, the net torque is finite and negative, indicating an intrinsic rotational bias and suggesting the presence of a preferred rotational state even in the absence of externally imposed twisting.

Movie captions.

Movie S1. Gliding behavior of afterfeather. Two afterfeathers with different handedness are placed inside a wind tunnel. Wind velocity: 0.05 m/s. Length of the afterfeather is 15 mm. The video is played at 4× acceleration.

Movie S2. Gliding behavior of afterfeather assemblies with different handedness. Wind velocity: 0.13 m/s. The video is played in real time.

Movie S3. Photo-induced deformation in right-handed afterfeathers and left-handed LCN assembly. UV: 385 nm, 200 mW/cm^2. Blue light: 460 nm, 157 m W/cm^2. The video is played at 2× acceleration.

Movie S4. Light switching of motion in right-handed afterfeathers and left-handed LCN assembly. UV: 385 nm, 150-200 mW/cm^2. Blue light: 460 nm, 100-170 mW/cm^2. The video is played at 4× acceleration.

Movie S5. Light switching of motion in right-handed afterfeathers and right-handed LCN assembly. UV: 385 nm, 150-200 mW/cm^2. Blue light: 460 nm, 100-170 mW/cm^2. The video is played at 4× acceleration.

Movie S6. Photo-induced deformation in right-handed afterfeathers and bending LCN assembly. UV: 385 nm, 200 mW/cm^2. Blue light: 460 nm, 157 mW/cm^2. The video is played at 2× acceleration.

Movie S7. Photo-controlled altitude inside the wind tunnel. UV: 385 nm, 150-200 mW/cm^2. Blue light: 460 nm, 100-170 mW/cm^2. The video is played at 4× acceleration.

Movie S8. Optical confinement along altitude direction. The flyer is positioned between two horizontally propagating light beams, with the UV beam above and the blue light beam below. UV: 385 nm, 200 mW/cm^2. Blue light: 460 nm, 157 mW/cm^2. The video is played at 16× acceleration.

Movie S9. Light selected take off among multiple assemblies. Various optical field patterns, i.e., round, bar, triangular and rectangular shapes are used to induce the opening of assembly to take off inside the wind tunnel. Light: white light from a projector, around 50 mW/cm^2 on the sample plane.

Movie S10. Collection of flyers using a gliding feather. A feather is positioned at the bottom of the wind tunnel to enable autonomous gliding. Different afterfeather assemblies are manually released from the top of the tunnel and collected by the feather below. The inlet wind velocity is 0.26 m/s. The video is shown at 4× speed.

Movie S11. CFD simulation of the pressure coefficient field evolution. The pressure coefficient distribution near the afterfeather-LCN assembly is shown as a function of time from

high-fidelity CFD simulations. The contours represent pressure coefficient, illustrating the flow structures in the vicinity of the feather. The views include the X-Z plane (top left), the X-Y plane (top right), and an isometric view (bottom left) with detailed visualization of the local flow features. The assembly structure is given at the bottom right. The video is played in real time.

REFERENCES

1. Y. Chen, H. Zhao, J. Mao, P. Chirarattananon, E. F. Helbling, N.-s. P. Hyun, D. R. Clarke, R. J. Wood, Controlled flight of a microrobot powered by soft artificial muscles. *Nature* **575**, 324–329 (2019).

2. Y.-H. Hsiao, A. Tagliabue, O. Matteson, S. Kim, T. Zhao, J. P. How, Y. Chen, Aerobatic maneuvers in insect-scale flapping-wing aerial robots via deep-learned robust tube model predictive control. *Sci. Adv.* **11**, eaea8716 (2025).

3. H.-V. Phan, D. Floreano, A twist of the tail in turning maneuvers of bird-inspired drones. *Sci. Robot.* **9**, eado3890 (2024).

4. V. Iyer, H. Gaensbauer, T. L. Daniel, S. Gollakota, Wind dispersal of battery-free wireless devices. *Nature* **603**, 427–433 (2022).

5. B. H. Kim, K. Li, J.-T. Kim, Y. Park, H. Jang, X. Wang, Z. Xie, S. M. Won, H.-J. Yoon, G. Lee, W. J. Jang, K. H. Lee, T. S. Chung, Y. H. Jung, S. Y. Heo, Y. Lee, J. Kim, T. Cai, Y. Kim, P. Prasopsukh, Y. Yu, X. Yu, R. Avila, H. Luan, H. Song, F. Zhu, Y. Zhao, L. Chen, S. H. Han, J. Kim, S. J. Oh, H. Lee, C. H. Lee, Y. Huang, L. P. Chamorro, Y. Zhang, J. A. Rogers, Three-dimensional electronic microfliers inspired by wind-dispersed seeds. *Nature* **597**, 503–510 (2021).

6. L. Ferreira Paiva, O. de Almeida, H. M. T. Silingardi, L. R. Novaes, Flight mechanics characteristics of wind-dispersed seeds from Brazilian savanna. *J. Braz. Soc. Mech. Sci. Eng.* **47**, 623 (2025).

7. F. Sui, W. Yue, K. Behrouzi, Y. Gao, M. Mueller, L. Lin, Untethered subcentimeter flying robots. *Sci. Adv.* **11**, eads6858 (2025).

8. S. Bai, Q. Pan, R. Ding, H. Jia, Z. Yang, P. Chirarattananon, An agile monopedal hopping quadcopter with synergistic hybrid locomotion. *Sci. Robot.* **9**, eadi8912 (2024).

9. S. Rafee Nekoo, R. Rashad, C. De Wagter, S. B. Fuller, G. d. Croon, S. Stramigioli, A. Ollero, A review on flapping-wing robots: Recent progress and challenges. *Int. J. Robot. Res.* **44**, 2305–2339 (2025).

10. B. Mazzolai, E. Del Dottore, T. Speck, N. Rowe, C. Laschi, Energy-saving movement strategies in animals and plants for robot design. *Nat. Rev. Bioeng.* **3**, 921–938 (2025).

11. J. M. Birch, M. H. Dickinson, Spanwise flow and the attachment of the leading-edge vortex on insect wings. *Nature* **412**, 729–733 (2001).

12. M. Seale, A. Kiss, S. Bovio, I. M. Viola, E. Mastropaolo, A. Boudaoud, N. Nakayama, Dandelion pappus morphing is actuated by radially patterned material swelling. *Nat. Commun.* **13**, 2498 (2022).

13. L. Sullivan, Deciding when to move. *eLife* **12**, e85477 (2023).

14. D. Wang, Z. Chen, M. Li, Z. Hou, C. Zhan, Q. Zheng, D. Wang, X. Wang, M. Cheng, W. Hu, B. Dong, F. Shi, M. Sitti, Bioinspired rotary flight of light-driven composite films. *Nat. Commun.* **14**, 5070 (2023).

15. J. Yang, H. Zhang, A. Berdin, W. Hu, H. Zeng, Dandelion-inspired, wind-dispersed polymer-assembly controlled by light. *Adv. Sci.* **10**, 2206752 (2023).

16. Y. Chen, C. Valenzuela, X. Zhang, X. Yang, L. Wang, W. Feng, Light-driven dandelion-inspired microfliers. *Nat. Commun.* **14**, 3036 (2023).

17. K. Johnson, V. Arroyos, A. Ferran, R. Villanueva, D. Yin, T. Elberier, A. Aliseda, S. Fuller, V. Iyer, S. Gollakota, Solar-powered shape-changing origami microfliers. *Sci. Robot.* **8**, eadg4276 (2023).

18. W. Yan, Y. Feng, J. Song, Z. Hong, K. Cui, A. C. Brannan, J. Tan, X. Song, Self-sensing dandelion-inspired flying soft actuator with multi-stimuli response. *Adv. Mater. Technol.* **9**, 2400952 (2024).

19. H. Mustafa, A. Nexha, T. Kister, H. Bartholomeus, T. Kraus, L. Kooistra, Spectroscopic characterization of laser-induced luminescence for remote environmental thermometry. *Opt. Express* **33**, 18492–18514 (2025).

20. B. Mazzolai, S. Mariani, M. Ronzan, L. Cecchini, I. Fiorello, K. Cikalleshi, L. Margheri, Morphological computation in plant seeds for a new generation of self-burial and flying soft robots. *Front. Robot. AI* **8**, 797556 (2021).

21. S. Arya, L. Spíchal, R. Zbořil, Flying seed-inspired sensors for remote environmental monitoring on Earth and beyond. *Trends Biotechnol.* **44**, 1214–1229 (2025).

22. F. Wiesemüller, Z. Meng, Y. Hu, A. Farinha, Y. Govdeli, P. H. Nguyen, G. Nyström, M. Kovač, Transient bio-inspired gliders with embodied humidity responsive actuators for environmental sensing. *Front. Robot. AI* **9**, 1011793 (2022).

23. C. Cummins, M. Seale, A. Macente, D. Certini, E. Mastropaolo, I. M. Viola, N. Nakayama, A separated vortex ring underlies the flight of the dandelion. *Nature* **562**, 414–418 (2018).

24. J. Yang, M. R. Shankar, H. Zeng, Photochemically responsive polymer films enable tunable gliding flights. *Nat. Commun.* **15**, 4684 (2024).

25. S. J. D. Lugger, S. J. A. Houben, Y. Foelen, M. G. Debije, A. P. H. J. Schenning, D. J. Mulder, Hydrogen-bonded supramolecular liquid crystal polymers: Smart materials with stimuli-responsive, self-healing, and recyclable properties. *Chem. Rev.* **122**, 4946–4975 (2022).

26. L. Xu, Y. Xu, L. Li, C. Qiu, Y. Ling, Y. Hu, L. Chang, Q. Peng, X. He, MXene-based soft actuators with phototropic self-sustained oscillation for versatile applications in micro robotics. *Adv. Funct. Mater.* **35**, 2414338 (2025).

27. C. Chen, Z. Liu, P. Shi, Y. Zhao, S. Duan, Y. Du, Y. Yan, M. Si, T. Iwasaki, X. He, Bio-inspired multimodal soft actuator with environmental self-adaptation. *Nat. Commun.* **16**, 7630 (2025).

28. J. M. McCracken, B. R. Donovan, T. J. White, Materials as machines. *Adv. Mater.* **32**, 1906564 (2020).

29. Z.-Z. Nie, B. Zuo, M. Wang, S. Huang, X.-M. Chen, Z.-Y. Liu, H. Yang, Light-driven continuous rotating Möbius strip actuators. *Nat. Commun.* **12**, 2334 (2021).

30. X. Zhao, Y. Chen, B. Peng, J. Wei, Y. Yu, A facile strategy for the development of recyclable multifunctional liquid crystal polymers via post-polymerization modification and ring-opening metathesis polymerization. *Angew. Chem. Int. Ed. Engl.* **62**, e202300699 (2023).

31. N. He, K. W. Kwan, A. H. W. Ngan, Enhancing the light-induced actuation of nickel oxyhydroxide by aluminum doping. *Adv. Mater. Technol.* **10**, 2401263 (2025).

32. J.-T. Kim, H.-J. Yoon, S. Cheng, F. Liu, S. Kang, S. Paudel, D. Cho, H. Luan, M. Lee, G. Jeong, J. Park, Y.-T. Huang, S. E. Lee, M. Cho, G. Lee, M. Han, B. H. Kim, J. Yan, Y. Park, S. Jung, L. P. Chamorro, J. A. Rogers, Functional bio-inspired hybrid fliers with separated ring and leading edge vortices. *PNAS Nexus* **3**, pgae110 (2024).

33. T. N. Sullivan, B. Wang, H. D. Espinosa, M. A. Meyers, Extreme lightweight structures: Avian feathers and bones. *Mater. Today* **20**, 377–391 (2017).

34. M. Lahikainen, H. Zeng, A. Priimagi, Reconfigurable photoactuator through synergistic use of photochemical and photothermal effects. *Nat. Commun.* **9**, 4148 (2018).

35. L. T. de Haan, J. M. N. Verjans, D. J. Broer, C. W. M. Bastiaansen, A. P. H. J. Schenning, Humidity-responsive liquid crystalline polymer actuators with an asymmetry in the molecular trigger that bend, fold, and curl. *J. Am. Chem. Soc.* **136**, 10585–10588 (2014).

36. R. V. Rohli, C. Li, “Pressure and winds,” in *Meteorology for coastal scientists*, R. V. Rohli, C. Li, Eds. (Springer International Publishing, 2021), pp. 139–145.

37. X. Zheng, Y. Jia, A. Chen, Azobenzene-containing liquid crystalline composites for robust ultraviolet detectors based on conversion of illuminance-mechanical stress-electric signals. *Nat. Commun.* **12**, 4875 (2021).

38. H. Guo, R. Nasare, C. Liang, K. Kuntze, E. M. Terentjev, A. Priimagi, Halogen-bonded liquid crystal elastomers as initiator-free photochemical actuators. *Adv. Mater.* **37**, e2504551 (2025).

39. W. Shen, J. Peng, R. Ma, J. Wu, J. Li, Z. Liu, J. Leng, X. Yan, M. Qi, Sunlight-powered sustained flight of an ultralight micro aerial vehicle. *Nature* **631**, 537–543 (2024).

40. K. Y. Ma, P. Chirarattananon, S. B. Fuller, R. J. Wood, Controlled flight of a biologically inspired, insect-scale robot. *Science* **340**, 603–607 (2013).

41. S. Kim, Y.-H. Hsiao, Z. Ren, J. Huang, Y. Chen, Acrobatics at the insect scale: A durable, precise, and agile micro–aerial robot. *Sci. Robot.* **10**, eadp4256 (2025).

42. S. Mariani, K. Cikalleshi, M. Ronzan, C. Filippeschi, G. A. Naselli, B. Mazzolai, A biodegradable, porous flier inspired by a parachute-like tragopogon fruit for environmental preservation. *Small* **21**, e2403582 (2025).

43. J. Yang, H. Pi, Z. Deng, H. Guo, W. Shou, H. Zhang, H. Zeng, Feedback regulated opto-mechanical soft robotic actuators. *Cell Rep. Phys. Sci.* **6**, 102686 (2025).

44. N. Dukhan, Forced convection of nanofluids in metal foam: An essential review. *Int. J. Therm. Sci.* **187**, 108156 (2023).

45. T. Esence, A. Bruch, S. Molina, B. Stutz, J.-F. Fourmigué, A review on experience feedback and numerical modeling of packed-bed thermal energy storage systems. *Sol. Energy* **153**, 628–654 (2017).
